# Machine learning based clinical decision tool to predict acute kidney injury and survival in therapeutic hypothermia treated neonates

**DOI:** 10.1038/s41598-025-01141-9

**Published:** 2025-05-19

**Authors:** Elif Keles, Syed Yaseen Ali, Pia Wintermark, Pieter Annaert, Floris Groenendaal, Suzan Şahin, Mehmet Yekta Öncel, Didem Armangil, Esin Koc, Malcolm R. Battin, Alistair J. Gunn, Adam Frymoyer, Valerie Chock, Djalila Mekahli, John van den Anker, Anne Smits, Karel Allegaert, Ulas Bagci

**Affiliations:** 1 Present Address: Department of Radiology, Feinberg School of Medicine, Northwestern University, 737 N. Michigan Avenue, Suite 1600, Chicago, IL 60611 USA; 2 Present Address: Division of Newborn Medicine, Department of Pediatrics, Montreal Children’s Hospital, Research Institute of the McGill University Health Centre, McGill University, Montreal, QC Canada; 3 Present Address: Department of Pharmaceutical and Pharmacological Sciences, KU Leuven, Leuven, Belgium; 4 Present Address: Department of Neonatology, Wilhelmina Children’s Hospital, University Medical Center Utrecht, and Utrecht University, Utrecht, The Netherlands; 5 Utrecht Brain Center, University Medical Center Utrecht, Utrecht, The Netherlands; 6 Present Address: Department of Neonatology, Faculty of Medicine, Izmir Demokrasi University, Izmir, Turkey; 7 Present Address: Department of Neonatology, Faculty of Medicine, İzmir Katip Çelebi University, İzmir, Turkey; 8 Present Address: Neonatal Intensive Care Unit, Koru Hospital, Ankara, Turkey; 9 Present Address: Department of Neonatology, Faculty of Medicine, Gazi University, Ankara, Turkey; 10 Present Address: Newborn Service, Auckland City Hospital, Health New Zealand, Auckland, New Zealand; 11 Present Address: Department of Physiology, University of Auckland, Auckland, New Zealand; 12 Present Address: Neonatal and Developmental Medicine, Stanford University School of Medicine, Palo Alto, CA USA; 13 Present Address: Department of Development and Regeneration, KU Leuven, Leuven, Belgium; 14 Department of Pediatric Nephrology, University Hospitals, Leuven, Belgium; 15 Present Address: Division of Clinical Pharmacology, Children’s National Hospital, Washington, DC USA; 16 Present Address: Neonatal Intensive Care Unit, University Hospitals Leuven, Leuven, Belgium; 17 Department of Hospital Pharmacy, Erasmus MC University Medical Center, Rotterdam, The Netherlands; 18 Department of Biomedical Engineering, Northwestern University, Chicago, USA; 19 Department of Electrical and Computer Engineering, Northwestern University, Chicago, USA

**Keywords:** Paediatric research, Translational research, Machine learning, Predictive medicine

## Abstract

Therapeutic hypothermia (TH) significantly reduces mortality and morbidities in neonates with Neonatal Encephalopathy (NE). NE may result in neonatal death and multisystem organ impairment, including acute kidney injury (AKI). Our study aimed to utilize machine learning (ML) methods to predict the outcome of TH-treated NE neonates developing AKI and death during TH. In this retrospective multinational study, 1149 TH-treated NE neonates and 801 controls were included. AKI was classified using KDIGO neonatal criteria based on serum creatinine measurements. The ML model incorporated gestational age, birth weight, postnatal age, and serum creatinine values. The algorithm used all these covariates to predict one of five outcomes: survival with/without AKI, mortality with/without AKI, and hospitalized non-NE controls. The XGBoost model achieved an AUC of 95% and an accuracy of 75.08% in predicting AKI and survival, surpassing other ML classifiers that demonstrated accuracy levels ranging from 54% to 65%. To our knowledge this is the first ML model trained on multicenter, multinational data specifically aimed at predicting neonates’ AKI, death, and survival within the first three days. Our ML scoring systems’ code and user interface are freely available (https://github.com/NUBagciLab/Therapeutic-Hypothermia-Outcome-Classification, https://thprediction.streamlit.app/). This tool has potential to support neonatologists to personalize therapies, and to optimize pharmacotherapy for renally cleared drugs.

**Supplementary Information:**

The online version contains supplementary material available at 10.1038/s41598-025-01141-9.

## Introduction

Birth asphyxia is characterized by a lack of oxygen with reduced brain blood flow around the time of birth, which can lead to Neonatal Encephalopathy (NE)^1^. NE is a clinical syndrome of neurologic dysfunction that encompasses a broad spectrum of symptoms and severity, from mild irritability and feeding difficulties to coma and seizures. The global prevalence of NE ranges from 1 to 3.5 per 1000 live births in high-income countries (HICs) to 26 per 1,000 in low- and middle-income countries (LMICs)^2,3^. In HICs, therapeutic hypothermia (TH) is an effective intervention that significantly reduces mortality and morbidities in neonates with moderate to severe NE^4^. However, NE is a multiorgan condition affecting more than just the central nervous system^5–7^.

The kidneys are highly vulnerable to oxygen deprivation, with acute kidney injury (AKI) occurring commonly (30–60%) as part of the “perinatal asphyxia syndrome,” now classified under the new kidney disease: Improving Global Outcomes (KDIGO) definition^8–12^. Growing evidence suggests that AKI is a significant risk factor for adverse long-term neurocognitive outcomes and increased mortality, longer hospitalization, and increased duration of mechanical ventilation^8,11,13^. Prompt diagnosis of AKI in TH-treated NE neonates is important both for renal management and to help identifying neonates who are most likely to have poor outcomes^8,14^. The neonatal modified KDIGO definition is currently the standard used in research and clinical practice. This definition is based on an increase in serum creatinine (sCr) or a reduction in urine output (UOP) (Table 1)^12^. However, the KDIGO criteria are difficult to apply in neonates because at birth sCr reflects maternal sCr and is already elevated. The typical physiological changes after birth involve fall in sCr over the first few weeks of life. The precise measurement of UOP can also be difficult and it is frequently low on the first day of life. Moreover, oliguria is not always present in neonates with AKI^15–17^. The definition of neonatal AKI is expected to evolve in the future. In our prior studies, we established the baseline serum creatinine values and changes in GFR and serum creatinine concentrations during TH for TH-treated NE neonates^18,19^. However, there is still an unmet clinical need to predict the clinical outcome of TH-treated NE neonates, including survival and AKI status.

**Table 1:** Modified neonatal kidney disease: improving global outcomes (KDIGO) criteria.

AKI stage	Serum creatinine (sCr) criteria	Urine output criteria (hourly rate)
0	No significant change in sCr or rise < 0.3 mg/dL	≥ 0.5 ml/kg/h
1	sCr rise by ≥ 0.3 mg/dL within 48 h or sCr rise ≥ 1.5-1.9x baseline sCr^a^	< 0.5 ml/kg/h x 6–12 h
2	sCr rise ≥ 2.0-2.9 x baseline sCr^a^	< 0.5 ml/kg/h for > 12 h
3	sCr rise ≥ 3 x baseline sCr^a^ or sCr ≥ 2.5 mg/dL^b^ or Kidney support therapy utilization	< 0.3 mL/kg/h for ≥24 h or Anuria for ≥ 12 h

^a^Baseline sCr defined as the lowest previous sCr value.

^b^sCr value of 2.5 mg/dL represents GFR < 10mL/min/1.73 m^2^. *sCr* serum creatinine, *h* hours. Adapted from KDIGO Acute Kidney Injury Workgroup^12^.

TH has been utilized to mitigate ischemic injury in kidney transplantation and after cardiopulmonary resuscitation but its impact on renal outcomes in neonates with NE is not well understood when NE has affected the kidney. The results of a meta-analysis of six TH trials of AKI in neonates with NE were inconclusive. A meta-analysis of six TH trials assessing the impact of TH on renal impairment did not find a statistically significant difference in the rate of renal impairment in cooled versus non-cooled neonates^4^. However, these studies were completed before the adoption of the KDIGO AKI classification for neonates, and the definitions of renal impairment varied among the studies^15^. A single center randomized controlled trial involving 120 term neonates with NE suggests that TH may reduce the risk of AKI (32% vs. 60%, *p* < 0.05)^20^.

Further research has assessed the effects of NE and TH on the glomerular filtration rate (GFR) and drug clearance related to GFR. Renal clearance was reduced by 25–40% and up to 60% as measured by mannitol clearance^21,22^. Except for one dataset on gentamicin clearance, most data have concentrated on the time when TH was being used (the first three days of life), with less evidence on later times. Thorough literature review revealed that increases in serum creatinine in this specific subpopulation were open to interpretation for the remainder of the first week of postnatal life^16^. It is also unlikely that additional studies comparing neonates with moderate to severe NE with and without TH would be conducted because TH has become the standard of care for NE treatment^8^. Supportive care should continue during TH, and further understanding drug clearance in neonates with NE is crucial because most medications, including antibiotics, inotropes, and antiepileptic drugs, are renally excreted^16,18,23^.

**Fig. 1:**
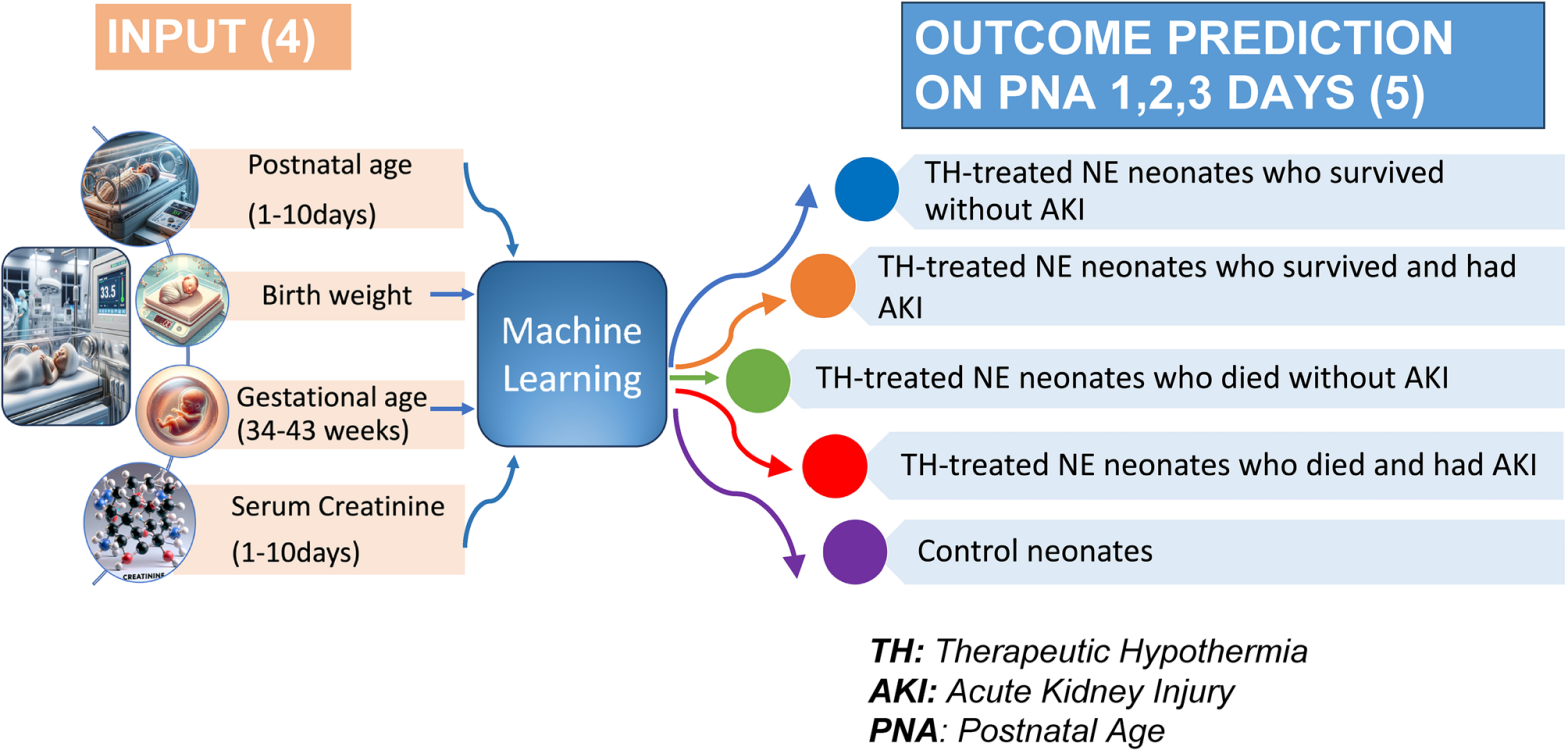
Graphical abstract of the newly developed model. Our algorithm was built on using four types of data: gestational age, birth weight, postnatal age, and serum creatinine observations during TH as input and predicting one of five classes as an outcome: (1) TH-treated NE neonates who survived, did not have AKI, (2) TH-treated NE neonates who survived and had AKI, (3) TH-treated NE neonates who died, did not have AKI; (4) TH-treated NE neonates who died and had AKI, (5) neonates without NE who did not need TH.

The overall goal of our study was to predict the outcome of TH-treated NE neonates regarding survival and AKI based on their gestational age (GA), birth weight, postnatal age (PNA), and serum creatinine measurements within the initial 10 days of life. In this study we therefore propose a new machine learning (ML) method to predict TH-treated NE neonates’ clinical outcome, including survival and associated AKI status during TH after postnatal day 1. Figure 1 shows our overall framework, utilizing an input with four parameters, and outcome prediction as the output.

## Results

All ML classifiers performed with an overall accuracy score (across all labels) of 54%–65% except for XGBoost, which predicted an outcome with 73% accuracy, which is why this classifier was selected to be further optimized (Table 2 and supplements).

**Table 2:** Machine learning classifiers and their explanations.

Employed models	Explanation	Accuracy score (%)
Logistic regression	Linear ML algorithm for probabilistic classification^51^. *StandardScalar*^52^ was used to ensure features on the same scale, a high maximum iteration parameter for convergence, and a random state for accuracy reproducibility	54
Random forest	It is based on decision tree algorithms, creating a ‘forest’ of trees where each tree makes its predictions when trained on a random subset of data^53^. These predictions are aggregated by a majority vote of the trees to decide the final output. Its ensemble nature helps avoid overfitting	63
Support vector classifier (SVC)	It works well in high dimensional spaces and is versatile because kernel functions transform feature space to fit different data distributions^54^. StandardScalar^52^ is used to scale the features and estimate the probabilities, which will use more internal cross validation^52^	58
Extreme gradient boosting (XGBoost)	Optimized distributed gradient boosting library that builds decision trees one at a time, with each new tree correcting the errors of the previous one in a greedy manner^36^. To assess classifier training loss, this implementation added an evaluation metric	73
Gradient boosting classifier	SciKit-Learn ensemble method that builds one tree. A random state is also used^55^	65
Adaptive boosting (AdaBoost)	Another ensemble method that fits a classifier on the dataset and then fits other classifiers on the dataset, adjusting the weights of incorrectly classified instances^56^	52
K-nearest neighbors (KNN)	The instance-based algorithm that stores all neonates and classifies new neonates by K-nearest neighbors^57^. StandardScalar^52^ was needed for feature scaling since KNN is distance-based	59
The decision tree	A nonparametric supervised learning method for classification and regression. Nodes are features, branches are decision rules, and leaves are outcomes. They tend to be overfit, but Random Forest and Extra Trees try to fix this^55^	56
Extra trees	Extremely Randomized Trees add a layer of randomness to bagging^55,58^. Extra trees can lead to a high variance and low bias model, which is beneficial for specific tasks	58
The multi-layer perceptron (MLP) neural network	A feed forward neural network^59^. They can capture complex relationships in data using multiple layers and nonlinear activation functions	60

The single classifier approach demonstrated superior precision and recall compared to the hierarchical classification approach, particularly for surviving neonates (Classes 1, 2 and 5) (Table 3).

**Table 3:** Performance metrics of hierarchical classification approach.

	TH-treated NE neonates who survived without AKI (Class 1)	TH-treated NE neonates who survived and had AKI (Class 2)	TH-treated NE neonates who died without AKI (Class 3)	TH-treated NE neonates who died and had AKI (Class 4)	Hospitalized (control-non NE) neonates (Class 5)
Hierarchical classification approach					
Precision	0.757	0.828	0.508	0.685	0.708
Recall	0.798	0.671	0.625	0.619	0.712
F1	0.777	0.740	0.560	0.647	0.737
Single classifier approach					
Precision	0.764	0.835	0.578	0.710	0.745
Recall	0.803	0.785	0.505	0.718	0.715
F1	0.783	0.809	0.537	0.708	0.729

In the hierarchical classification approach, precision scores ranged from 0.508 to 0.828, with lower precision for TH-treated NE neonates who died without AKI. Recall varied from 0.619 to 0.798, with TH-treated NE neonates who survived without AKI having the highest recall, F1-scores reflect a generally balanced performance (Table 3).

The single classifier strategy demonstrated a stronger precision relative to the hierarchical method, achieving a precision of 0.835 for TH-treated NE neonates who survived and had AKI. Recall varied between 0.505 and 0.803, with lower recall for TH-treated NE neonates who died without AKI. The F1-scores show that TH-treated NE neonates who survived and had AKI (0.809) exhibited the highest classification performance (Table 3). This single classifier provided the highest predictive capability, reiterated by precision, recall and F1 score (Table 3).

The first classifier in the hierarchical model struggled to predict TH treated NE neonates who died with or without AKI with recall, precision and F1 scores of the death cases ranging from 48%–56%, while in the single classifier model, they ranged between 51%–58% (Table 3). The only notable decrease observed with the single classifier model compared to the hierarchical model was in the ‘TH-treated NE neonates who died without AKI’ label, which had about a 12% decline in recall score. Despite this, the overall performance showed significant improvements. The classification performance improved across multiple labels, with some showing minor gains and others significant enhancements, leading to an overall increase in accuracy, precision, recall, and F1 scores (Table 3).

**Fig. 2:**
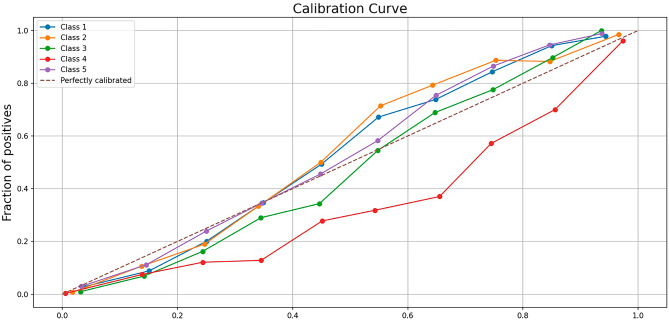
Calibration curve of our single classifier approach model. This plot shows the calibration of the predicted probabilities across five classes (Class 1 to Class 5). The x-axis represents the mean predicted probability, while the y-axis represents the fraction of positives (observed frequency). Each line corresponds to a different class, with the dashed diagonal line (“Perfectly calibrated”) indicating an ideal calibration where predicted probabilities perfectly match the observed outcomes. Deviation from the diagonal suggests miscalibration, where probabilities either underestimate or overestimate the actual outcomes.

**Fig. 3:**
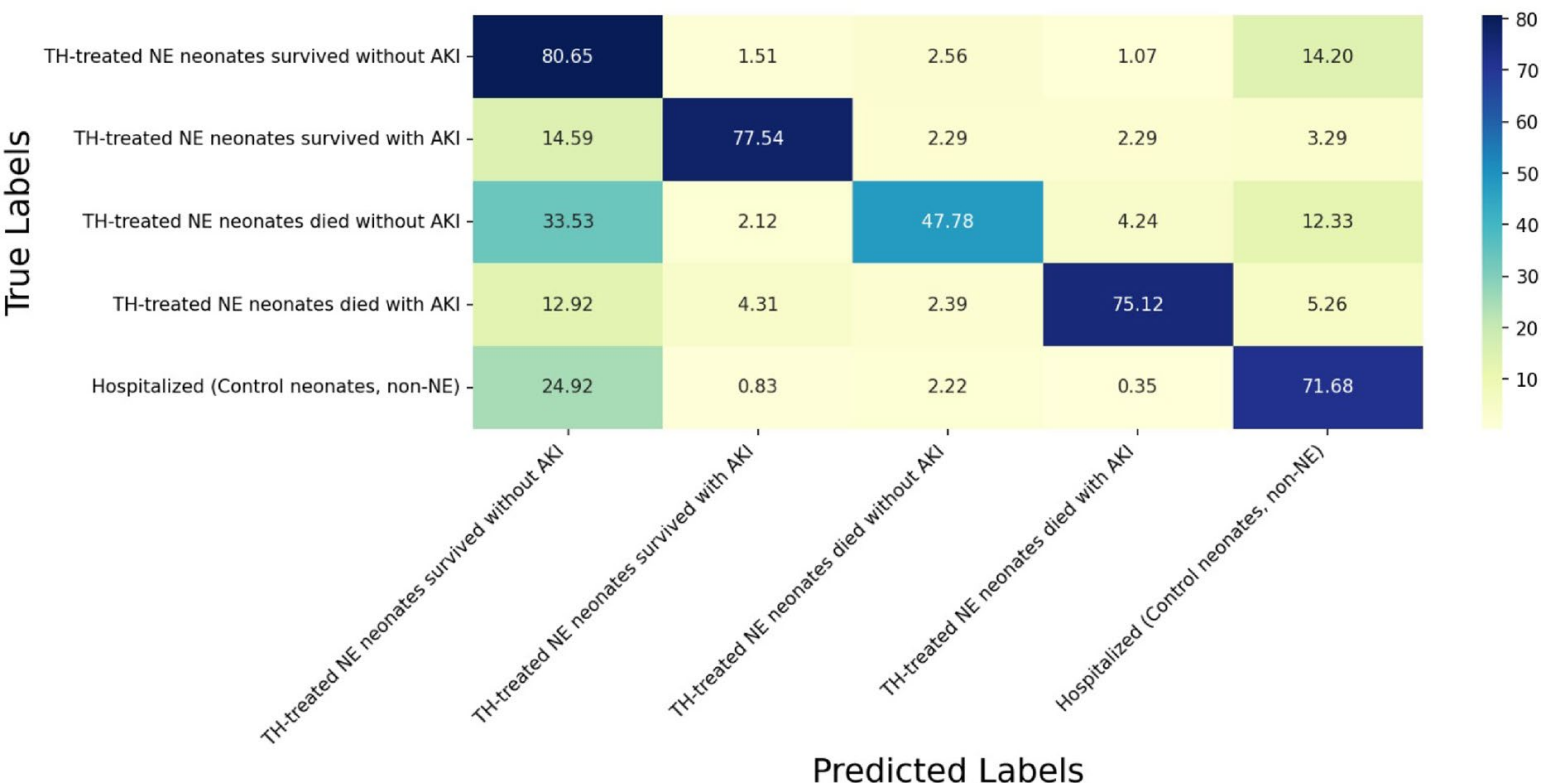
Confusion matrix of our developed model. The confusion matrix illustrates the percentage of cases correctly predicted by our model. The y-axis represents the true labels, while the x-axis represents the predicted labels. The bold boxes highlight the percentage of true labeled patients correctly predicted by the model. For instance, the model correctly predicted “TH-treated NE neonates survived without AKI” in 80.65% of cases.

The classification of neonates who died without AKI had the lowest performance in both approaches, highlighting challenges to correctly identify this group. By contrast, TH-treated NE neonates who survived and had AKI had the best classification results across both approaches. Hospitalized (Control-non-NE) neonates were classified with relatively high scores in both models (Table 3; Figs. 2 and 3, Supplement).

**Fig. 4:**
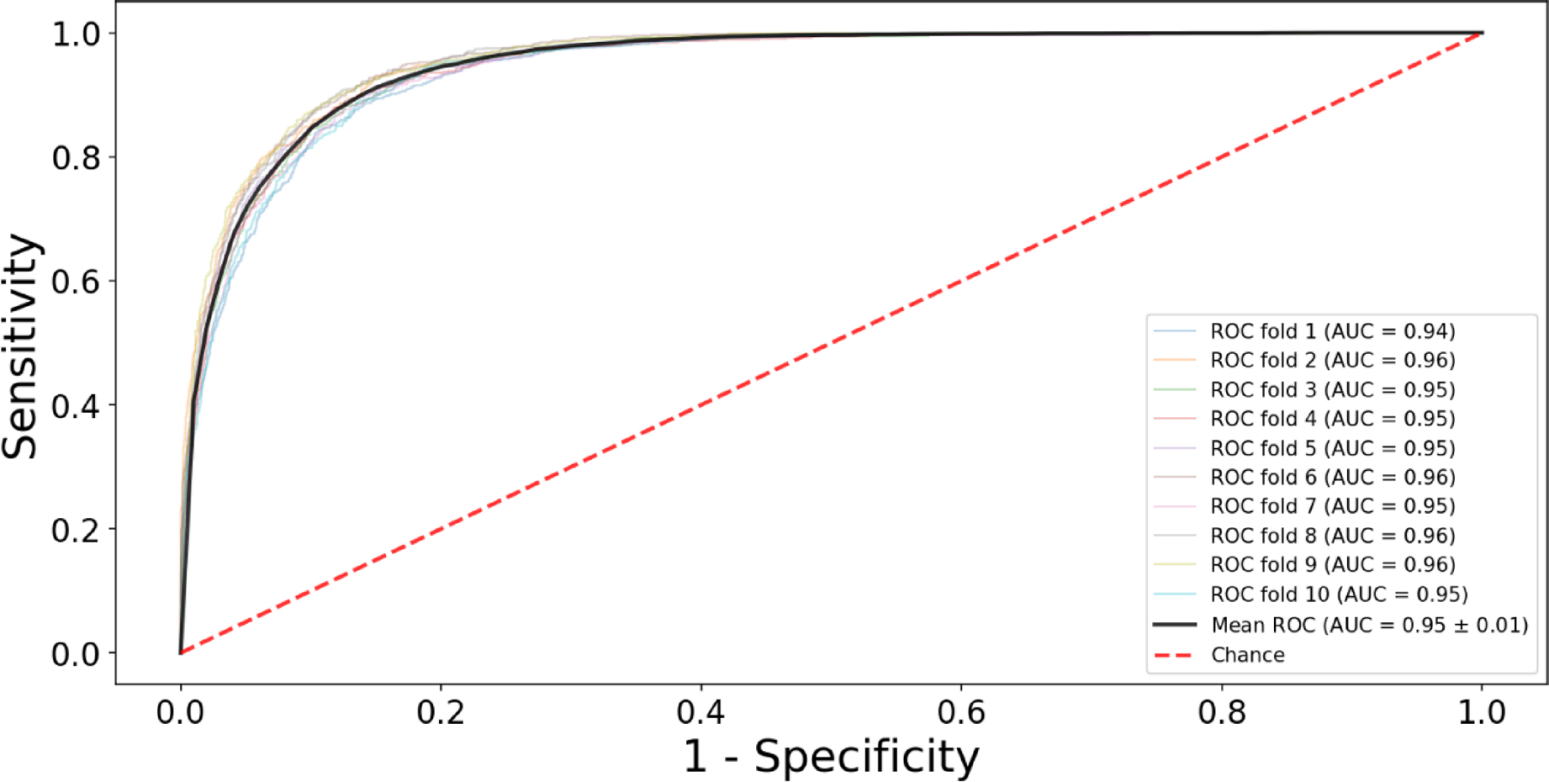
ROC of our model predicting clinical outcome in TH-treated neonates. ROC graphs for our model, cross-validated using ten-fold validation. During each of the ten folds, the model undergoes training and testing, and the Area Under the Curve (AUC) is computed for each iteration. The ten AUC values are combined to yield a singular performance metric through averaging. The model’s mean AUC of 95% over all 10 folds demonstrates its constant and excellent performance in accurately differentiating between classes.

Beyond accuracy, precision, recall and F1 scores, our model demonstrated excellent classification with a mean AUC of 0.95 ± 0.01 across 10-fold cross-validation. This indicates that the model has a high probability of correctly distinguishing between different outcomes in TH treated NE neonates (Fig. 4). The model becomes slightly more accurate as folds of cross-validation increase, indicating a continuous model improvement with an increase in training data, while simultaneously confirming the model’s generalizability. From 5, 10, to 20 folds of cross-validation, we observed 74.2%, 75.1%, and 75.1% accuracy, respectively. All the curves are close together and have high AUCs, which means the model performed consistently (Fig. 4). The calibration curve for the single classifier model showed good performance, except for class 4 (infants who died with AKI).

Our newly developed model demonstrates promising potential for predicting outcomes in TH treated NE neonates as evidenced by the confusion matrix, the model exhibits high accuracy in predicting survival with and without AKI and death with AKI. The matrix also highlights areas for improvement, particularly in predicting outcomes for TH treated NE neonates died without AKI. It originated from class imbalance (Fig. 3).

To further assess the model’s performance, the Matthews correlation coefficient (MCC) was calculated based on the confusion matrix. For our five-class classification, the overall MCC is 0.656, indicating a moderately strong positive correlation between the predicted and actual class labels (Table 4). Classes 2 and 4 which refer to TH-treated NE neonates survived with AKI and TH-treated NE neonates who died with AKI) have high MCC values (0.797 and 0.786). This suggests that the model is remarkably effective at identifying neonates with AKI compared to all other outcomes. Class 1 (TH-treated NE neonates who survived without AKI) had a lower MCC (0.502), and Class 3 (TH treated NE neonates who died without AKI) had a moderate MCC (0.571). Class 5, the control group (hospitalized neonates, non-NE), showed an MCC of 0.614, which is moderate. This result suggests the model is performing effectively, accurately classifying many instances across all classes.

**Table 4:** Matthews correlation coefficient (MCC) values for single classifier model and all 5 classes.

Overall single classifier models’ MCC	0.656
Class 1 (TH treated NE neonates survived without AKI)	0.502
Class 2 (TH treated NE neonates survived with AKI)	0.797
Class 3 (TH treated NE neonates died without AKI)	0.571
Class 4 (TH treated NE neonates died with AKI)	0.786
Class 5 (Hospitalized control neonates, non NE)	0.614

**Fig. 5:**
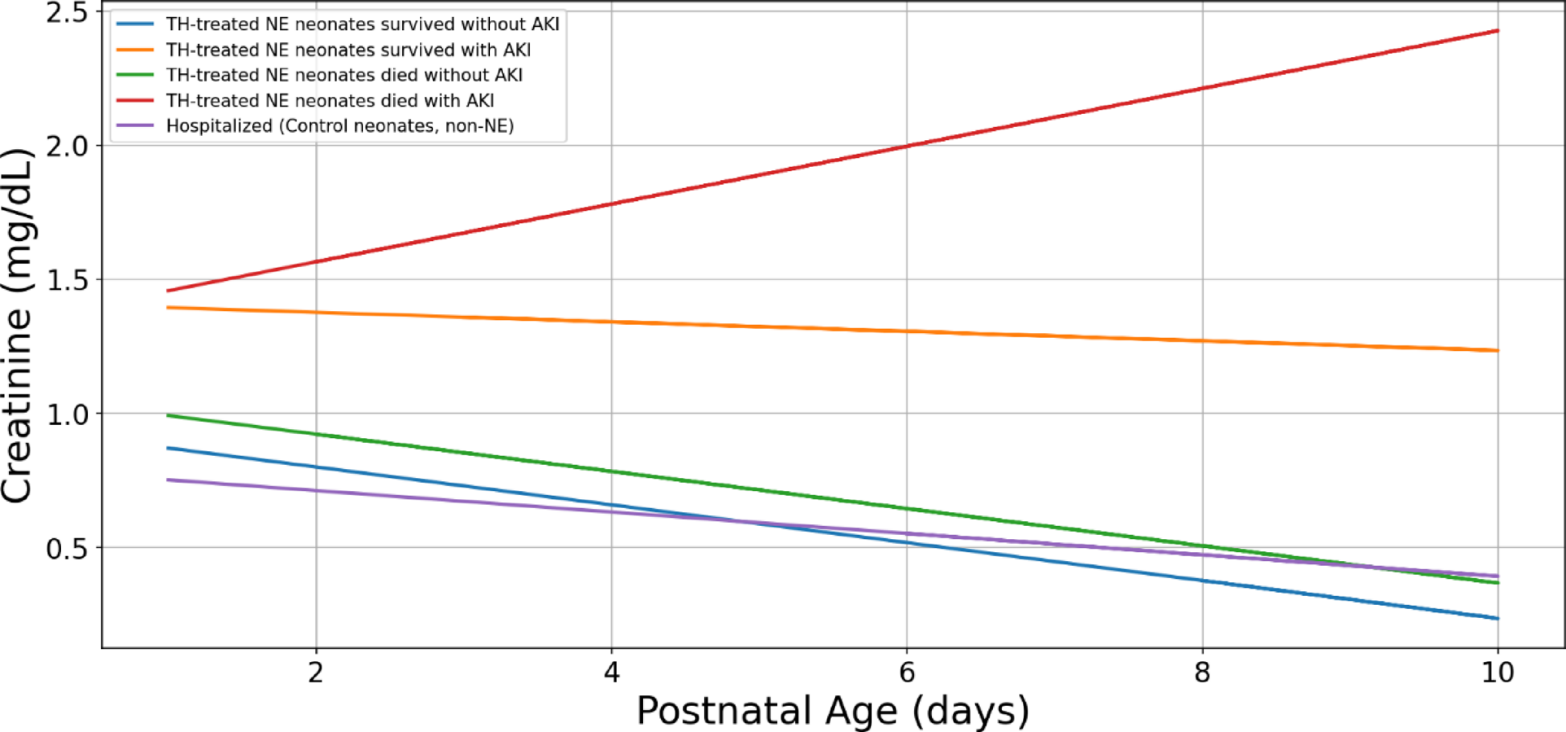
Serum Creatinine concentrations for different patient groups in our model. The graph displays the simplified median trend lines creatinine (sCr) observations for each label in our model over a period of 10 days. These are actual values from the model. The baseline sCr values for each labeled group are shown, along with their trends over time.

**Fig. 6:**
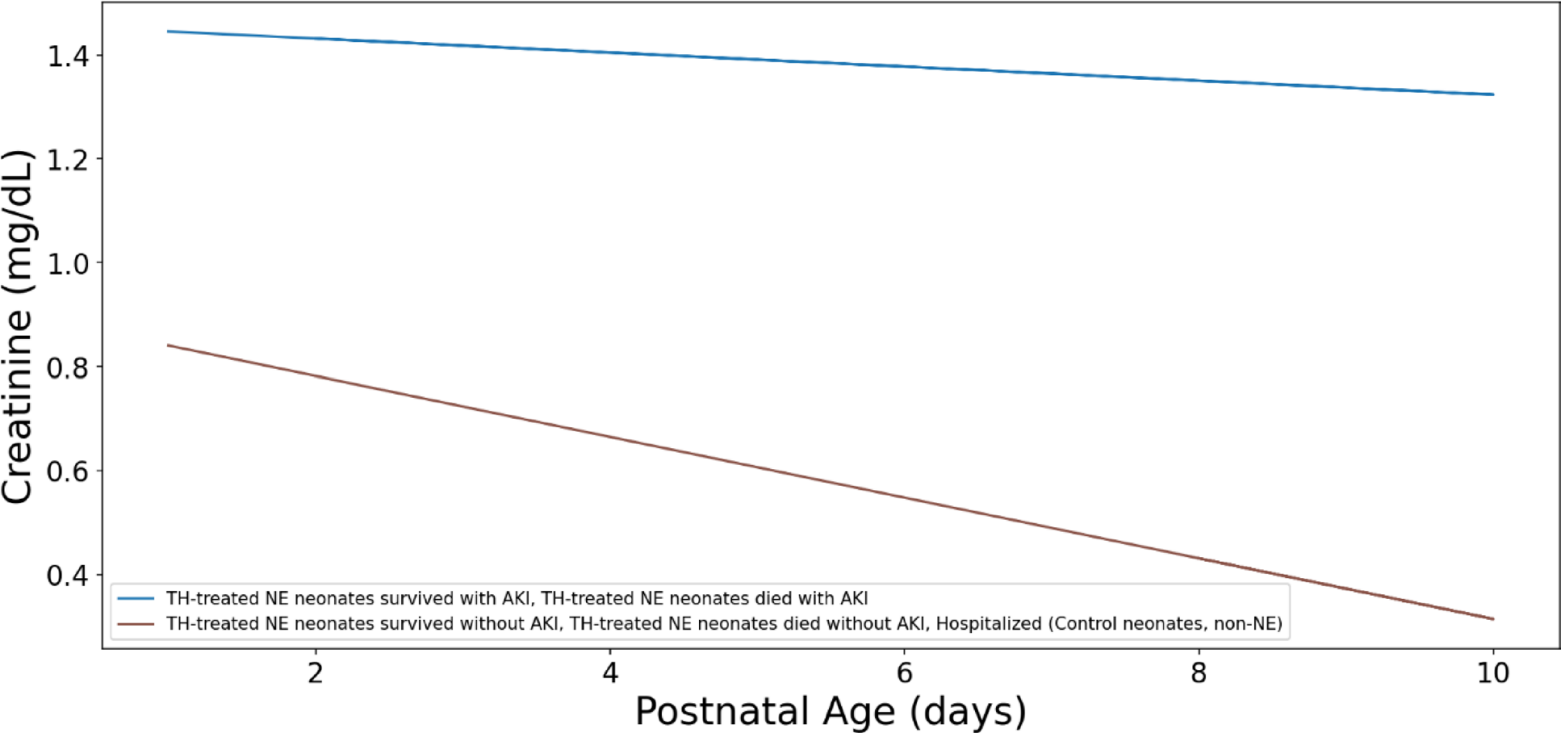
Serum creatinine concentrations of neonates with AKI and without AKI. The graph illustrates the median creatinine (sCr) trends of neonates with AKI (both those who survived and those who died) and neonates without AKI. The neonates without AKI are further divided into three groups: hospitalized non-NE neonates, neonates who died, and neonates who survived.

We then examined the creatinine trends to understand the explainability of our models. On the first day, all TH-treated neonates had very similar sCr values. However, in the following days, these values diverged, reflecting different outcomes. Interestingly, the serum creatinine trends for TH-treated NE neonates who survived without AKI closely mirrored those of hospitalized control neonates until day 3 (Figs. 5 and 6).

Next, we analyzed the serum creatinine trends for TH-treated NE neonates with AKI, irrespective of survival status, along with TH-treated neonates without AKI and hospitalized control neonates (Fig. 5). The graph shows two distinct trend lines for serum creatinine: higher creatinine, stable trend and lower creatinine, decreasing trend. In the AKI groups, serum creatinine concentrations started higher and remained relatively stable over the 10-day period. This suggests persistent kidney injury in this group. In neonates without AKI, serum creatinine concentrations started lower and gradually decreased over time, indicating improving kidney function (Figs. 5 and 6). The divergence of these two trends was apparent early on, suggesting that serum creatinine concentrations are a valuable early indicator of AKI in NE neonates (Figs. 5 and 6).

**Fig. 7:**
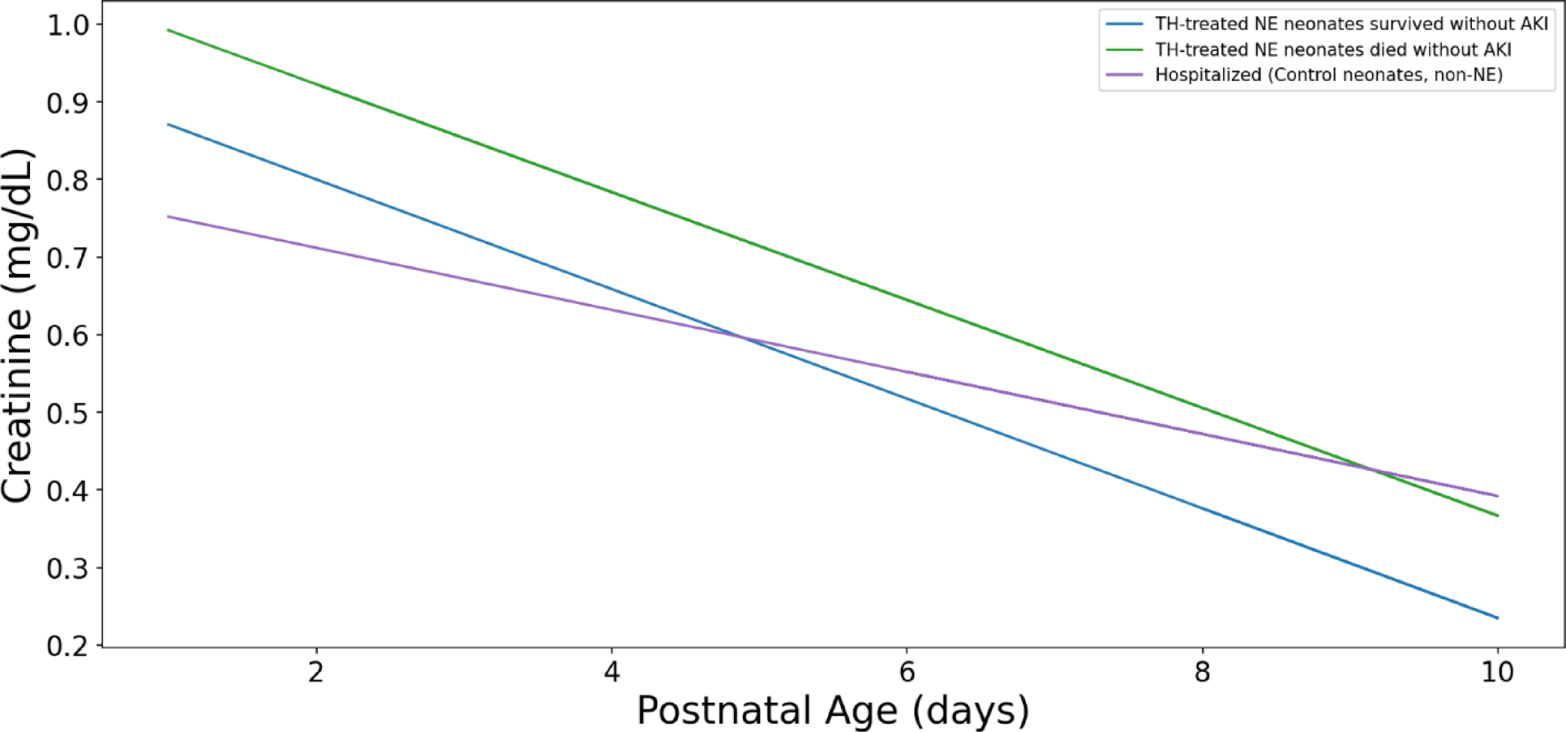
Serum creatinine concentrations for all TH-treated NE neonates and control neonates. The graph shows the median creatinine (sCr) trend lines for neonates who did not have AKI, divided into three groups. The creatinine trends for these groups overlapped during their NICU stay, and the median creatinine values were close to each other. This illustrates the difficulty in differentiating between these groups based solely on creatinine values.

**Fig. 8:**
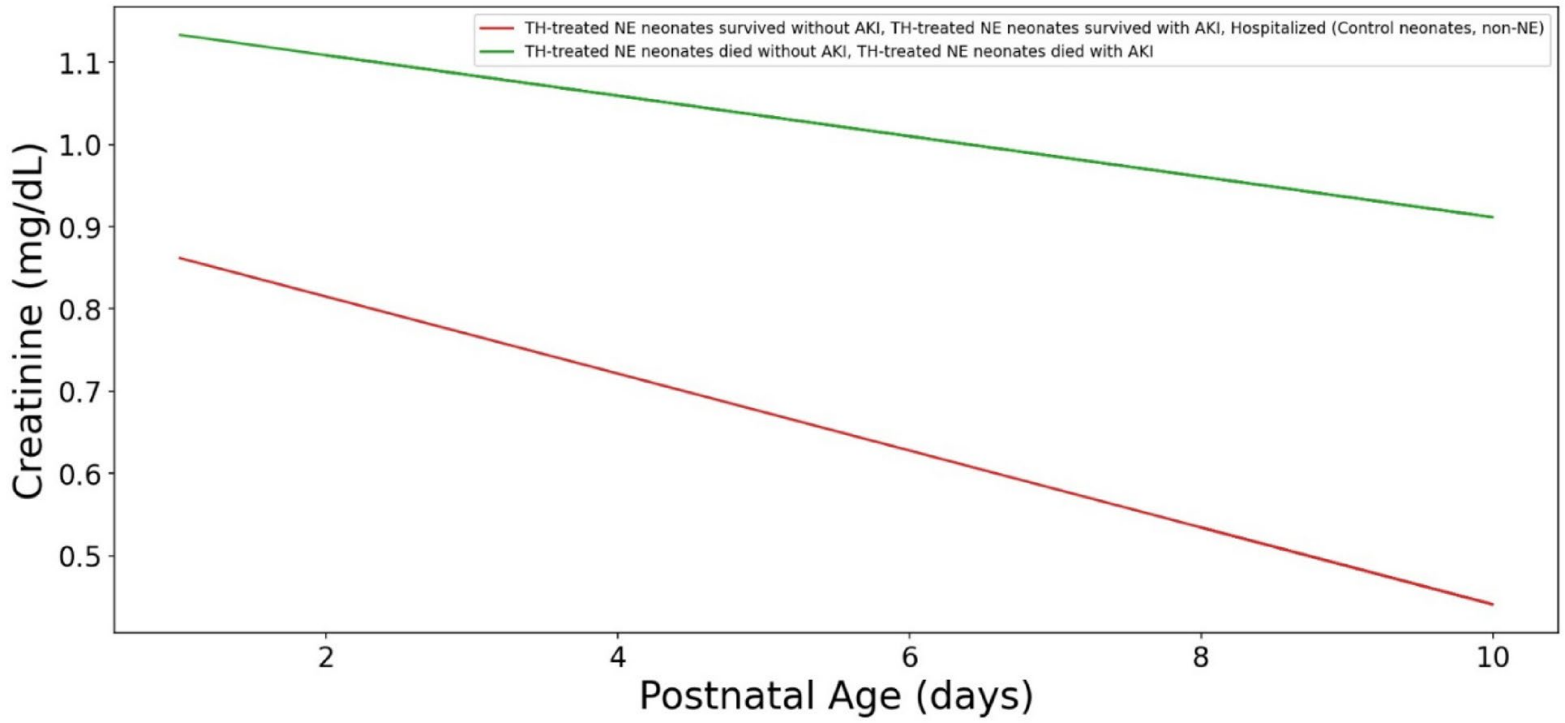
Serum creatinine concentrations for all TH-treated NE neonates who survived and who died in the dataset. The graph illustrates the median creatinine (sCr) levels for neonates who survived and those who died. The trend lines show that survived neonates had lower creatinine levels compared to those who died.

We closely examined serum creatinine trends in the non-AKI groups and showed that serum creatinine trends overlapped during their NICU stay (Fig. 7). We also closely examined neonates who deceased and those who survived. The trend lines show that surviving neonates had lower serum creatinine concentrations as compared to those who passed away (Fig. 8).

**Fig. 9:**
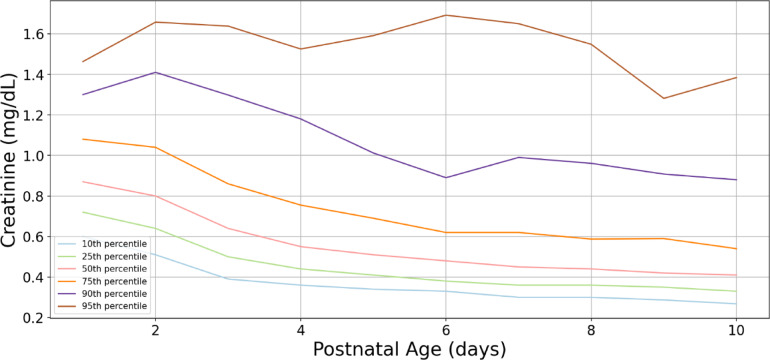
Percentiles of serum creatinine concentrations based on postnatal age on the study dataset. The graph shows the percentiles of serum creatinine (sCr) concentrations based on postnatal age for the entire study dataset. The median centile lines illustrate the sCr values for both NE neonates and hospitalized non-NE neonates.

**Fig. 10:**
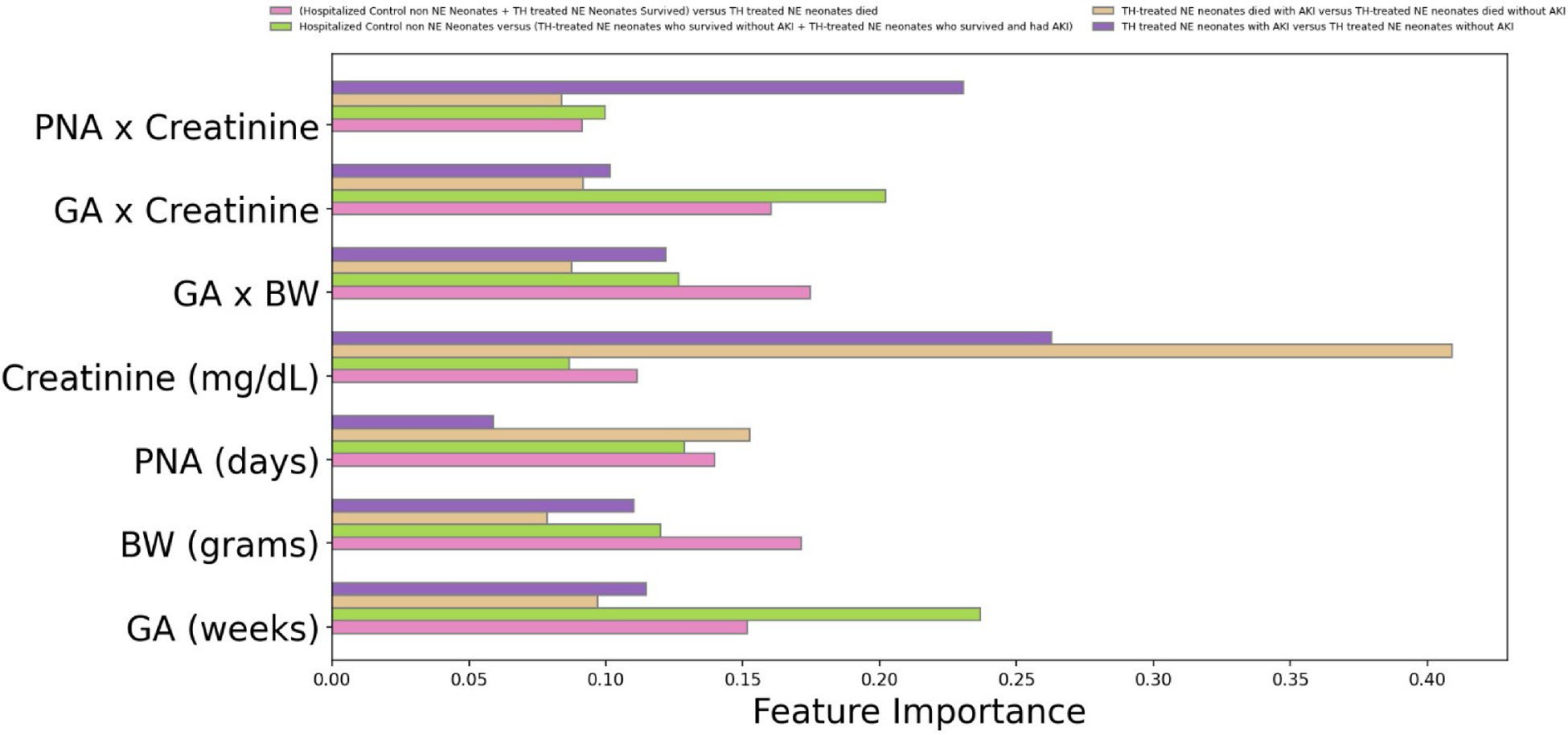
Features’ importance of each classifier in the hierarchical classification model. The ranking of features (variables) plays a crucial role in predicting outcomes for a hierarchical classification model. Pink, green, pastel orange, and purple lines represent each of the outcome predictions. Input variables interact with each other for the outcome prediction. Creatinine, postnatal age interaction, and creatinine play important roles in algorithmic decision-making.

**Fig. 11:**
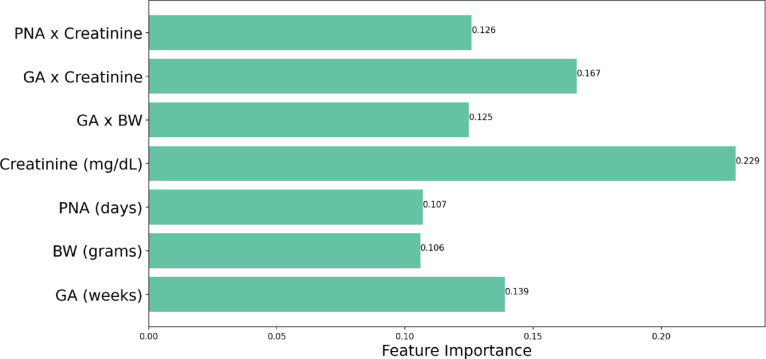
Features’ importance of the single classifier model approach. Ranking the importance of features in predicting outcomes using a “single classifier model”. The relationship between creatinine and gestational age is crucial in the decision-making process of algorithms.

Consistent with our previous research^19^ we report serum creatinine centile lines for all our data including TH treated NE neonates and hospitalized non-NE neonates (Fig. 9). To understand the model’s decision-making black box nature, we undertook feature importance analysis for both hierarchical and single classifier models for each class. Serum creatinine, and postnatal age interaction with serum creatinine play important roles in the hierarchical algorithmic decision-making (Fig. 10). Feature importance in single classifier model revealed serum creatinine and gestational age–creatinine interaction in decision making (Fig. 11). Our analysis revealed no linear relationship between the input variables. Instead, the models capture complex, nonlinear interactions that are critical for accurate predictions. These interactions are essential for the decision-making mechanism of the models, as they reflect the complex nature of physiological processes in neonates.

## Discussion

AKI has been associated with worse outcomes in previous studies, including increased mortality, prolonged length of stay, increased need for mechanical ventilation, and more adverse neurocognitive outcomes^8,16,24^. It is still difficult to rapidly assess renal function in this high-risk group in order to detect AKI and start suitable treatment promptly. Neonatologists focus on detecting AKI based on the sCr concentrations, urine output, urine biomarkers, and noninvasive near-infrared spectroscopy (NIRS) monitoring^8^. A study in of 53 neonates using blood biomarkers from the beginning of TH achieved AUC by 0.61 in 2 h of life for serum NGAL (neutrophil gelatinase-associated lipocalin) suggesting this has acceptable accuracy to identify developing AKI^25^. Another study of 110 NE neonates combined serum and urine biomarkers to predict AKI from the 24 h of life. Among those markers a urinary NGAL achieved an AUC of 0.86 to predict AKI^26^. Urine biomarkers were collected within the 12, 24, 48, and 72 h of life in 64 TH treated NE neonates^27^. In this study urine KIM-1 (Kidney Injury Molecule-1) had an AUC of 0.79 at 48 h of life. Renal oxygen saturations were higher in the AKI group than non-AKI NE neonates, and renal saturation > 75% achieved an AUC of 0.76 within the 48 h of life^28^. However, these methods are not practical or clinically adapted for early detection of AKI and other outcomes^8^. Our algorithm predicts AKI and death in TH-treated NE neonates with an AUC of 95% and an accuracy of 75.08% for AKI and survival, outperforming urine and blood biomarkers in predictive accuracy^26,27^.

The present study highlights that diagnosing AKI in neonates requires a comprehensive approach that cannot solely rely on serum creatinine concentrations. Meticulous clinical monitoring can significantly enhance detection accuracy. Integrating diagnostic tools with clinical decision-making enables healthcare professionals to more effectively identify AKI, facilitating timely interventions that can improve patient outcomes. Given the limited availability of urine and blood biomarkers in practice in the NICU, our approach would not only ensure accurate diagnosis but also help guide evidence-based treatment decisions, ultimately enhancing patient care and improving outcomes.

At present, there are only two alternative neonatal and pediatric calculators to estimate risk of AKI, using Logistic regression methods, which are the Baby NINJA^29^ and STARZ^30^ calculator. Those two calculators use electronic health records (EHR). Baby NINJA is a warning system to decrease nephrotoxic events based on the EHR^29^. The STARZ-Neonatal AKI risk stratification uses 10 predictors to predict the onset of AKI. STARZ was designed using 744 neonates’ data, including postnatal age at NICU, serum creatinine, sepsis, use of PPV (positive pressure ventilation), inotropes, urine output, furosemide use, the status of cardiac disease, gestational age and nephrotoxic drugs^30,31^. Neither were developed to predict AKI in TH-treated NE neonates. Advancements in neonatal care have decreased the mortality of TH-treated NE neonates. However, the daily management of the multiorgan failure of these neonates, especially during the first days of life, remains important to help limit other morbidities. We expect that our tool will help physicians reduce the morbidities of TH-treated NE neonates. Our user interface will be available for the public and for research use to help predict clinical outcomes. Based on “gestational age, birth weight, postnatal day (within the first three days) and serum creatinine value (within the first three days)”, the system will give the potential clinical outcomes with probabilities.

Although the present algorithm achieved 95% of AUC, the reader should consider some limitations. Most recent developments in artificial intelligence rely on deep learning, which has become a game changer for many research fields, from computer science to healthcare^32^. The dataset defines which model could be suitable for the algorithm^32^. Deep learning typically requires much larger datasets, even if our dataset is the largest multicenter dataset described to date in this context^32,33^. Our output is categorical, and the number of variables for each patient is limited for evaluating classifier performance in unbalanced datasets, traditional metrics like accuracy can be misleading. Instead, metrics such as precision, recall, F1-score, and confusion matrices provide a clearer picture of model effectiveness by considering both false positives and false negatives. Ensuring robust model evaluation is essential for developing reliable classification systems in clinical settings^32,34^. Although the overall accuracy of 73% may appear modest, it must be interpreted in the context of complex, multi-class clinical outcomes. The inclusion of sensitivity, specificity, precision, and related metrics provides a nuanced evaluation and helps establish clinically acceptable thresholds. The Matthews correlation coefficient (MCC) offers a more comprehensive evaluation by accounting for all aspects of classification errors, providing a more robust and clinically meaningful interpretation of model performance^35^.

Our model performed well in terms of Matthews correlation coefficient. No previous study has used any biomarker or predictive tool to obtain these outcomes with these specific metrics. For survivors without AKI (Class 1), the model achieves a high F1-score (0.783), reflecting strong precision and recall. However, the Matthews correlation coefficient (MCC) is notably lower (0.502), suggesting that while the model identifies many true positives, it may also misclassify a substantial number of non-Class 1 cases. This discrepancy indicates that F1 may overestimate performance in this scenario, as it does not account for true negatives (Tables 3 and 4).

In contrast, for survivors with AKI (Class 2), the alignment between F1 (0.809) and MCC (0.797) suggests a well-balanced classification with minimal misclassification errors. This consistency indicates that the model effectively identifies this outcome without introducing systematic bias toward a particular class (Tables 3 and 4). For neonates who died without AKI (Class 3), both F1 (0.537) and MCC (0.571) indicate moderate classification performance. While precision and recall remain suboptimal, the slightly higher MCC suggests a more favorable overall error distribution when considering the complete confusion matrix (Tables 3 and 4).

For neonates who died with AKI (Class 4), F1 (0.708) is moderate; however, MCC (0.786) is substantially higher. This indicates that although direct detection performance is reasonable, the overall classification—accounting for true negatives and error distribution—is stronger. In a clinical context, where distinguishing high-risk cases is critical, this robustness is particularly relevant (Tables 3 and 4).

For hospitalized control neonates (Class 5), F1 (0.729) exceeds MCC (0.614), suggesting that while precision and recall are favorable, the model’s overall classification performance diminishes when true negatives are considered. This again highlights the tendency of F1 to overestimate performance by underrepresenting misclassified negative cases (Tables 3 and 4).

These observations underscore the importance of using MCC alongside the F1-score for medical classification tasks. The discrepancies, particularly in Class 1 and Class 5, suggest that models may overestimate performance when evaluated solely on F1. Conversely, the alignment of MCC and F1 in Class 2 supports confidence that the model is providing balanced performance. In high-risk cases such as Class 4, the high MCC suggests that the model effectively differentiates these outcomes from others, even when precision and recall are only moderate.

Our current approach accounts for temporal trends through strategic data partitioning. However, it does not fully leverage the sequential nature of time-series data. Variables such as serum creatinine and postnatal age exhibit dynamic fluctuations that may be more effectively captured using sequential deep learning architectures, including recurrent neural networks, long short-term memory networks, or temporal convolutional networks. While dataset size precluded the implementation of these methods in the present study, future research should explore their potential to enhance temporal modeling and improve predictive performance in clinical applications.

A decline in performance between the hierarchical and single classifiers was observed in TH-treated NE neonates who died without AKI. This may be attributed to overlapping clinical features among subgroups and inherent model limitations in detecting subtle biomarker variations. Additional inputs, clinical variables may be necessary to enhance subgroup distinction and improve model performance. These lower numbers may indicate that the features used to characterize neonates without AKI—whether they survived or died—are less distinctive or more overlapping with those of other classes. Even though hospitalized non-NE controls share some similarities with certain NE groups in baseline clinical characteristics, making the classification task more challenging.

We encountered a data imbalance problem during algorithm development. We have started to develop a hierarchical model of four XGBoost^36^ classifiers that make binary classification decisions in cascading order. To increase model performance, further oversampling methods and interaction factors were introduced. However, the model’s performance was not comparable or superior. Due to multiple attempts and combinations of methodologies, this model may not be improved. Data imbalance is a common issue in clinical machine learning that can negatively impact classification performance^37–40^. Addressing this challenge requires tailored approaches, including data-level, algorithmic, and ensemble methods^37–40^. Data-level techniques such as under-sampling and over-sampling, including SMOTE, help balance datasets, while hybrid methods combine these strategies for improved representation. In clinical applications, where datasets often contain healthier individuals than affected cases, these strategies improve detection in disease diagnosis. Integrating sampling techniques with algorithmic adjustments yields optimal results, though deep learning approaches require substantial computational resources. Future research should refine these strategies to enhance model generalizability in medical classification tasks.

There are also different methods to handle data imbalance problems, but their selection should be optimized according to the dataset and clinical scenario. In our predictive algorithm to address clinical questions, we used known predictors and aimed to fit them into a statistically viable algorithm. This has never been done before. Since we are already limited to certain predictors, we tried to utilize them to explore non-linear relationships between the variables, and to predict the patient outcome based on this non-linear relation. Different classifiers, even the same classifiers with different kernels (such SVM examples), the classifiers will behave differently. Based on what we observe in the behavior, we optimized all selected classifiers.

To address class imbalance, we applied a random oversampling technique; however, we acknowledge that this manual oversampling may not generalize across different clinical datasets. Moreover, our feature set—limited to gestational age, birth weight, postnatal age, and serum creatinine—may not capture the full complexity of neonatal outcomes. Additional factors, such as markers of hypoxic-ischemic encephalopathy (HIE) severity, inflammatory biomarkers, and resuscitation details, could further enhance model performance. Future studies should aim to incorporate these variables and explore more sophisticated oversampling or augmentation techniques.

Our input selection was guided by the availability and established clinical relevance of gestational age, birth weight, postnatal age, and serum creatinine. We acknowledge that additional factors—such as the severity of hypoxic-ischemic encephalopathy (HIE), the presence of PPHN, sepsis, neonatal resuscitation details, and other biomarkers—may significantly impact outcomes. These variables could not be used in the present analysis due to missing data. Future studies should integrate these predictors to enhance model performance and clinical applicability.

The serum creatinine centile trends were very similar, making it challenging to distinguish among these three groups based solely on creatinine values. This suggests that serum creatinine alone may not be the most reliable predictor of outcomes in these specific groups of neonates (Figs. 3 and 5).

Artificial intelligence implementations in pediatrics and neonatology have drawn attention to decision-making and clinical support systems^32^. Here, we performed an ML analysis on a multicenter international retrospective cohort of TH-treated NE neonates. Due to the data scarcity in pediatrics and neonatology^32^, a natural difficulty in such fields, we put our effort to train and test our algorithm on the largest dataset ever. We followed statistical evaluation paradigms (cross validation) to avoid any bias in evaluations. For reproducibility and generalizability, we share our code in GitHub and user interface publicly.

Further research is needed to evaluate the prospective use of our model. It is highly likely that its performance could be enhanced by incorporating additional variables and larger datasets.

Although sCr has its limitations as a biomarker of kidney function and injury, serial measures of sCr over the first week of life can help establish a pattern of renal function. We used the neonatal KDIGO definition to diagnose AKI, neonatal KDIGO is the most used standard definition for AKI. Our dataset consists of neonates from 1999 to 2021. The use of TH has likely improved over time and includes both selective head cooling to total body cooling. The dataset does not include details on demographics and pharmacotherapy during NICU.

As a collaborative team of neonatologists and AI scientists, we emphasize the critical role of clinical decision support systems and the importance of ‘human-in-the-loop’ approaches in medical applications. Tools like our prediction model offer clinicians valuable, objective data on AKI and survival status, while preserving clinician autonomy in the final decision-making process. As clinical decision support systems become more integral to medical practice, our tool addresses a crucial gap, particularly during the critical first 72 h of life for neonates undergoing TH.

We developed our model based on the first 10 days of life. With our supervised ML model, the model was able to predict clinical outcomes within the first 3 days with an AUC of 95%. In addition to careful monitoring of clinical parameters, this clinical decision tool might tailor future physiologically based therapeutic approaches or support precision medicine decisions. By providing insights into potential organ injury, our model encourages timely consideration of AKI and survival outcomes, potentially enabling earlier renal-protective interventions like methylxanthines^41^ and improving survival rates for affected neonates.

## Methods

This study reanalyzed previously reported pooled datasets on sCr^18,19^ (Tables 5 and 6). The initial study protocol was approved by the Ethics Committee Research of UZ /KU Leuven (S63365). Informed written consent was hereby waived. We confirm that all research was performed in accordance with relevant guidelines/regulations.

**Table 5:** Description of the cohorts of TH-treated NE neonates included in the pooled study^19^.

Cohort	Time interval	Characteristics	Observations	Count of creatinine observations	Therapeutic hypothermia criteria
Leuven	2010–2020	TH-treated NE neonates in whom amikacin pharmacokinetics (Leuven, Amsterdam) were reported were included^43^	87 neonates 355 sCr assay: Jaffe, to enzymatic	1–10	In the first 6 h, and (i) gestational age (GA) ≥ 36 weeks, (ii) at least one asphyxia condition: Apgar_5min_ ≤ 5, *or* need for resuscitation or respiratory support in the first 10 min, *or* umbilical cord pH < 7.0 with a base deficit ≥ − 16 mmol/L, or lactate > 10 mmol/L within the first hour, and (iii) signs of NE (Thompson ≥ 7, or amplitude-integrated electroencephalography, aEEG)^42,44^
		This cohort was extended to all TH-treated NE neonates admitted to Leuven unit during the time interval	13 Neonates 82 sCr assay: enzymatic	2–8	
CoolCap	1999–2002	The objective of the CoolCap study was to determine whether 72 h mild TH provided by selective head cooling, started within 6 h, improved survival and neurodevelopmental outcome at 18 months in neonates with moderate or severe NE^45^. Only TH-treated NE neonates were included	111 neonates 439 sCr assay: both, but unknown, center-specific	1–9	(i) GA ≥ 36 weeks, (ii) at least one asphyxia condition: Apgar_5min_ ≤ 5, *or* continued need for resuscitation *or* respiratory support at 10 min after birth, *or* umbilical cord pH < 7.0 or a base deficit >− 16 mmol/L, or in an arterial/venous sample within the first hour, and (iii) moderate to severe NE consisting of altered state of consciousness (shown by lethargy, stupor, or coma) AND at least one or more of the following: hypotonia, abnormal reflexes, absent or weak suck, clinical seizures, and (iv) abnormal background aEEG (20 min)^45^
Zekai Tahir Burak	2011–2014	In a prospective nested case-control study, data in TH-treated NE neonates were provided^46^	40 neonates 80 sCr assay: Jaffe	2	TH initiation was based on the Total Body TH for Perinatal Asphyxia (TOBY) criteria^46^
Ankara	2015–2021	Koru Hospital^19^	82 neonates 493 sCr assay: Jaffe	3–12	Asphyxia was defined by (i) Apgar_5/10min_ ≤5; (ii) cord blood pH < 7.00 or BE of ≥-16 mmol/L, further confirmed by (ii) imaging evidence of NE-compatible brain injury or multiorgan failure^60^. Neonates (GA ≥ 36 weeks) with perinatal asphyxia were eligible within 6 h if they had NE (Thompson > 5, Sarnat Stage 2 or 3, discontinuous regular voltage pattern or worse on aEEG, or convulsions. Neonates with known congenital deformities or disorders were excluded. For patients who did not meet all diagnostic criteria (neonates 34–35 weeks GA, patients with a cord blood gas pH > 7 and a BE between − 12 and − 16), TH initiation was discussed with the consultant^60^
	2013–2021	Gazi University Hospital^19^	54 neonates 488 sCr assay: Jaffe	3–26	
Montreal	2008–2021	sCr observations reported (*n* = 202, 2009–2015)^61^ were further extended	439 neonates 2171 sCr assay: enzymatic	1–31	TH criteria were (i) GA ≥ 36 weeks and birth weight ≥ 1800 g, (ii) evidence of fetal distress, i.e. history of an acute perinatal event, cord pH < 7.0 or base deficit >− 16 mEq/L, (iii) evidence of neonatal distress, such as an Apgar_10min_ ≤ 5, postnatal blood gas pH obtained within the first hour ≤ 7.0 or base deficit >-16 mEq/L or continued need for ventilation initiated at birth and continued for at least 10 min, and (iv) evidence of moderate to severe NE indicated by abnormal neurological exam and/or aEEG^61^
Stanford	Various	Pooling of 3 previously reported studies^14,28,47^	63 neonates 457 sCr assay: both (study specific)	1–16	≥ 36 weeks GA diagnosed with moderate or severe NE underwent TH. TH criteria were as outlined in the National Institute of Child Health and Human Development (NICHD) TH study^62^
Utrecht	2008–2021	Data extraction from the medical files in all TH-treated NE neonates^19^	260 neonates 961 sCr assay: enzymatic	1–11	TH inclusion criteria in the first 6 h were (i) GA ≥ 36 weeks, (ii) at least one asphyxia condition: Apgar_5min_ ≤ 5, *or* need for resuscitation or respiratory support in the first 10 min, *or* cord blood pH < 7.0 with a base deficit >− 16 mmol/L, or lactate > 10 mmol/L within the first hour, and (iii) clinical signs of NE (Thompson ≥ 7) or aEEG background abnormalities (discontinuous normal voltage with a baseline below 5 µV) or seizures^44^

**Table 6:** Description of the control cohort of hospitalized neonates included in the pooled study^48^.

Cohort	Time interval	Characteristics	Observations	Count of creatinine observations
Leuven	2007–2011	This cohort included 1080 neonates admitted to NICU (24–42 weeks of GA) over the first 6 weeks of PNA^48^ GA ≥ 36 weeks neonates included Diverse clinical indications (including suspected infections, respiratory conditions, congenital anomalies) TH-treated NE neonates were excluded	801 controls 2881 sCr observations during the first 10 days Assay: enzymatic	1–16

### Datasets in TH-treated NE neonates and non-TH-treated, non-NE control neonates

Data from 8 TH-treated NE cohorts were combined^19^ (Table 5). The first ten days of PNA were analyzed to assess recovery of kidney function over time. Day 1 was hereby defined as the date of delivery (from birth until 24 h). AKI detection was based on the KDIGO definition (any AKI): sCr↑ ≥0.3 mg/dL within 48 h or sCr↑ ≥1.5 fold versus the lowest prior sCr within 10 days, irrespective of urine output. Data on NE severity, fluid management, perinatal pharmacotherapy, comorbidity, or urine output were not available. sCr observations in controls were extracted from an already published sCr population model as a time-dependent covariate in neonates^14,19,28,42–48^. The covariates collected in both datasets were restricted to birth weight, GA, survival (neonatal death, day 1–28, yes/no), and sCr values (day 1–10) to facilitate pooling. We have 1149 TH treated NE neonates with 5526 sCr observations within the first 10 days. Information on included cohorts of TH-treated NE neonates is provided in Table 5.

We analyzed serum creatinine (sCr) data from 801 control neonates with a GA of ≥ 36 weeks and postnatal age (PNA) of 1–10 days, collected from the neonatal intensive care unit of the University Hospitals Leuven (2007–2011) (Table 6). Neonates treated for infections, respiratory adaptation, and congenital malformations were included, excluding those receiving therapeutic hypothermia. The dataset yielded 2,881 sCr measurements within the first 10 days and relevant covariates (birth weight, GA).

The descriptive statistics of covariates for TH-treated NE neonates and control neonates are presented in Tables 7 and 8. The sCr values obtained by the Jaffe assay were converted to values equivalent to ones obtained by an isotope dilution mass spectrometry (IDMS) traceable enzymatic assay using the following Eq. (1): sCr_IDMS_ = 1.003 × sCr_Jaffe_ + 0.057, (conversion, 1 mg/dL = 88.4 µmol/L).

**Table 7:** Neonates’ number for each group in dataset.

Patient status	Number of observations
TH-treated NE neonates survived without AKI	4099
TH-treated NE neonates survived with AKI	699
TH-treated NE neonates died without AKI	519
TH-treated NE neonates died with AKI	209
Hospitalized (control neonates, non-NE)	2881
Total	8407

**Table 8:** Patient characteristics of NE and control neonates.

Groups and clinical variables	Minimum	Maximum	Median	Mean
TH-treated NE neonates				
Body weight (g)	1750	6230	3340	3347.81
Postnatal age (days)	1	10	3	3.36
Creatinine (mg/dL)	0.08	4.1	0.74	0.84
Gestational age (weeks)	34	43	39	39.03
Control neonates				
Body weight (g)	1280	6000	3167.5	3154.34
Postnatal age (days)	1	10	4	4.34
Creatinine (mg/dL)	0.18	4.13	0.57	0.62
Gestational age (weeks)	35	42	38	38.27

### Machine learning (ML)

The general basis of the ML model was to use the four types of neonatal data available in our multicenter international pooled dataset (GA, birth weight, PNA, and creatinine values) as input and to determine one of the five classes of outcomes that were described in our dataset, which are:

1. TH-treated NE neonates who survived without AKI.
2. TH-treated NE neonates who survived with AKI.
3. TH-treated NE neonates who died without AKI.
4. TH-treated NE neonates who died and had AKI.
5. Hospitalized neonates who did not need TH.

As the outcome of the prediction is categorical, a classification model would be ideal for our needs. Different classifiers were experimented to figure out the most capable model to engineer and yield the most optimal results^49^ (Table 1 and Supplements). These models include Logistic Regression, Random Forest, Support Vector Classifier (SVC), Extreme Gradient Boosting (XGBoost), Gradient Boosting, Adaptive Boosting (AdaBoost), K-Nearest Neighbors (KNN), Decision Tree Classifier, Extra Trees Classifier, and a neural network^49^.

In this study, a hierarchical model of four XGBoost classifiers was proposed as a simplification in the decision-making process of the classifier, as each classifier would be responsible for binary classification, and decisions would be made in cascading order, from the broadest decision to the eventual selection of a label, in a similar fashion to a decision tree. This model was further optimized using parameter optimization through *GridSearch*, interaction features derived from the input data, and oversampling of the classes where TH resulted in mortality since there was a large imbalance between survived and death cases of treatment. Oversampling was handled using the *RandomOverSampler* from the imbalance learning package (*imblearn*)^50^. Although the oversampling did improve the classification of predicting neonatal deaths from day 1 remained the model’s overall weakness.

We take considerable steps to prevent data leaking and ensure that our model’s performance is accurate and applicable to new data.

#### Patient-level splitting

During the cross-validation procedure, we separated data from each patient. All data points (serum creatinine values) from a single patient were assigned to either the training or test set, never both. This ensures that the model does not “learn” from the same patient’s data in both sets by accident.

We used a method called stratified K-fold (*StratifiedKFold*) cross-validation, which ensures that the balance of groups is kept in each split.

Despite all this model tuning, an overall accuracy of no more than 73.5% was obtained. This is due to an expected drop-off in accuracy, even after tuning, when faced with cascading decision processes. Furthermore, the model was severely limited as the first and broadest classifier in the model, which predicted between alive or dead neonates, suffered the lowest accuracy due to data imbalance, which is survived neonates’ number are more than dead neonates (Table 7), ultimately capping the model’s performance at the level of its weakest classifier.

A reversion to a single XGBoost classifier predicting all five labels was made to negate this phenomenon of cascading error. Interaction features and *GridSearch* were also applied with slightly different parameters to account for differences in the model. Oversampling was programmed once again using *RandomOverSampler*, which functions manually this time by selecting the minority labels, specifying the desired sample count for the minority labels, then continuously randomly sampling from the minority classes until the ideal amount is reached and concatenating that set to the dataset for training. This was done since techniques such as the Synthetic Minority Oversampling Technique (SMOTE) yielded mediocre results. Patient-Level Splitting *and* Stratified Cross-Validation (*StratifiedKFold)* were once again used for testing. We employed a double cross-validation, wherein the model underwent k-fold cross-validation. Additionally, the dataset utilized comprised one of five stratified datasets, each with its corresponding test set, which was entirely excluded from the training process. After testing the respective training sets with their corresponding test sets, the results were averaged to obtain the current accuracy. This approach offers greater protection against bias for the model than traditional cross-validation methods. The model achieved an overall accuracy score of 75.1%. In evaluating classification performance, key metrics such as accuracy, precision, recall, F1-score, and AUC provide essential insights, each with distinct strengths and limitations (Table 9).

**Table 9:** Evaluation metrics in machine learning.

$$\:\text{Accuracy}=\frac{TP+TN}{TP+TN+FP+FN}$$
$$\:{F}_{1}=2\times\:\frac{\text{Precision}\times\:\text{Recall}}{\text{Precision}+\text{Recall}}$$
$$\:\text{Precision}=\frac{TP}{TP+FP}$$
$$\:\text{Recall}=\frac{TP}{TP+FN}$$

Accuracy quantifies the proportion of correctly classified instances across all predictions but can be misleading in imbalanced datasets, where a model may achieve high accuracy by predicting only the majority class while failing to detect minority cases. Where true positives (TP) and true negatives (TN) represent correctly classified instances, while false positives (FP) and false negatives (FN) denote misclassified cases (Table 9).

While accuracy is intuitive, it has notable limitations, particularly in imbalanced datasets where one class significantly outweighs another. A model could achieve high accuracy simply by predicting the majority class while failing to detect the minority class altogether. In such scenarios, additional metrics like precision, recall, F1-score, and ROC-AUC are essential to provide a more comprehensive evaluation of model performance (Table 9).

‘Overall accuracy score’ is defined as the proportion of correctly predicted cases relative to the total number of cases. This metric provides an initial measure of model performance across all outcome classes, although its limitations in the context of imbalanced data are acknowledged. Accuracy indicates the overall proportion of correct predictions; AUC measures the model’s discriminative ability; MCC offers a balanced performance measure even with imbalanced classes; and precision, recall, and F1-scores provide insight into the true positive rates and error margins.

F1 Score is the harmonic mean of precision and recall. Detailed definitions were given below (Table 9).

AUC (Area Under the Curve) evaluates the classifier’s ability to distinguish between classes, with higher values indicating better discrimination. It remains useful even in imbalanced datasets, as it considers performance across varying decision thresholds.

A combination of these metrics is essential for a robust evaluation, particularly in medical classification tasks where both false positives and false negatives carry significant consequences.

MCC (Matthews Correlation Coefficient), in a one-vs‐all setting, incorporates all four elements of the confusion matrix (TP, TN, FP, FN). It is therefore more balanced, especially when there is an uneven distribution of negatives versus positives. MCC values range from − 1 to + 1, where + 1 represents perfect prediction, 0 reflects random prediction, and − 1 signifies complete disagreement between predictions and actual outcomes.

We also looked at the calibration curves of our single classifier models (Fig. 9). To assess our model’s performance on the imbalanced dataset, we calculated the Matthews correlation coefficient (MCC)^35^. Recent research and applications highlight the robustness of MCC in multi-class problems, particularly in domains where class distributions are highly skewed. Studies suggest that MCC provides more reliable insights than metrics that may disproportionately favor the majority class.

This methodology benefited our model incorporating post-natal age (PNA) as a variable and utilizing multiple measurements per patient enabled the model to analyze the temporal variations of creatinine levels in relation to other clinical factors. We also inserted the creatinine percentiles within first ten days.

### Comprehensive evaluation of classification performance

Our models were trained using four input variables: gestational age (GA), birth weight (BW), post-natal age (PNA), and creatinine values (Cr). The feature analysis was conducted to enhance the explainability of our models and to understand the interaction among these variables. We employed feature importance techniques and interaction bars to illustrate how these variables contribute to the model’s predictions.

### User interface for practical application

We developed an intuitive user interface integrated with the final version of the trained ML model (XGBoost); code was made publicly available too (https://github.com/NUBagciLab/Therapeutic-Hypothermia-Outcome-Classification). The model takes input from all the metrics it was trained on (gestational age, birth weight, postnatal age, and creatinine level), with the option to choose between mg/dL and µmol/L for creatinine levels. When the “Predict” button is pressed, the model receives the input, which is used to predict the outcome TH (https://thprediction.streamlit.app/). It prints out one of five outputs, each message representing one of the five possible outcomes, followed by a confidence interval (probabilistic score). A confidence interval was implemented within the prediction model, which will provide us with a plausible range of an estimate to express the uncertainty of said estimate. This implementation involved a 100 bootstrap sample dataset to calculate the mean probability of the predicted outcome. Then, the standard deviation of the samples is computed to measure the variability in predictions, and the confidence interval is calculated using the 95% CI formula, where 95% of data lies within 1.96 standard deviations of the mean.

Odds ratios were also calculated to measure the association between exposure and an outcome, specifically the relative odds of death given the presence of AKI. This was calculated by first finding the odds of death in the case of infants with AKI and then the odds of death without AKI. The odds ratio is the ratio of these two odds, comparing the likelihood of death in patients with AKI against those without AKI (Figures in Supplements).

## Electronic supplementary material

Below is the link to the electronic supplementary material.

Supplementary Material 1

## Data Availability

User interface and code are freely and publicly available (https://github.com/NUBagciLab/Therapeutic-Hypothermia-Outcome-Classification, https://thprediction.streamlit.app/). Dataset is available from the corresponding author upon reasonable request.
