# Supplementary material for: Machine learning based clinical decision tool to predict acute kidney injury and survival in therapeutic hypothermia treated neonates
Source: Sci Rep. 2025 May 19;15:17278. doi: 10.1038/s41598-025-01141-9 (PMC12089479; doi:10.1038/s41598-025-01141-9)
Supplement: Supplementary file 1 — Supplementary Material 1 [file 41598_2025_1141_MOESM1_ESM.docx]

| **Classifier** | **TH-treated NE neonates who survived without AKI**  **Precision** | **TH-treated NE neonates who survived without AKI Recall** | **TH-treated NE neonates who survived without AKI TH F1** | **TH-treated NE neonates who survived and had AKI Precision** | **TH-treated NE neonates who survived and had AKI Recall** | **TH-treated NE neonates who survived and had AKI**  **F1** | **TH-treated NE neonates who died without AKI**  **Precision** | **TH-treated NE neonates who died without AKI**  **Recall** | **TH-treated NE neonates who died without AKI**  **F1** | **TH-treated NE neonates who died and had AKI**  **Precision** | **TH-treated NE neonates who died and had AKI**  **Recall** | **TH-treated NE neonates who died and had AKI**  **F1** | **Hospitalized (Control-non NE) neonates**  **Precision** | **Hospitalized (Control-non NE) neonates**  **Recall** | **Hospitalized (Control-non NE) neonates**  **F1** | **Model Accuracy** |
| --- | --- | --- | --- | --- | --- | --- | --- | --- | --- | --- | --- | --- | --- | --- | --- | --- |
| **Logistic**  **Regression** | 0.553 | 0.767 | 0.643 | 0.524 | 0.355 | 0.424 | 0 | 0 | 0 | 0.5 | 0.067 | 0.118 | 0.523 | 0.411 | 0.46 | 0.543 |
| **Random**  **Forest** | 0.644 | 0.743 | 0.69 | 0.667 | 0.579 | 0.62 | 0.326 | 0.149 | 0.204 | 0.636 | 0.311 | 0.418 | 0.635 | 0.607 | 0.621 | .634 |
| **SVC** | 0.579 | 0.769 | 0.661 | 0.564 | 0.349 | 0.431 | 0 | 0 | 0 | 1 | 0.067 | 0.125 | 0.592 | 0.526 | 0.557 | .583 |
| **XGBoost** | 0.717 | 0.804 | 0.758 | 0.857 | 0.75 | 0.8 | 0.721 | 0.307 | 0.431 | 0.844 | 0.6 | 0.701 | 0.715 | 0.704 | 0.709 | .730 |
| **Gradient Boosting** | 0.642 | 0.794 | 0.71 | 0.711 | 0.533 | 0.609 | 1 | 0.109 | 0.196 | 0.64 | 0.356 | 0.457 | 0.648 | 0.6 | 0.623 | .651 |
| **AdaBoost** | 0.536 | 0.742 | 0.622 | 0.59 | 0.388 | 0.468 | 0 | 0 | 0 | 0.304 | 0.378 | 0.337 | 0.47 | 0.331 | 0.388 | .515 |
| **KNN** | 0.6 | 0.724 | 0.656 | 0.558 | 0.474 | 0.512 | 0.308 | 0.079 | 0.126 | 0.571 | 0.267 | 0.364 | 0.588 | 0.542 | 0.564 | .588 |
| **Decision Tree** | 0.626 | 0.626 | 0.626 | 0.503 | 0.539 | 0.521 | 0.248 | 0.257 | 0.252 | 0.36 | 0.4 | 0.379 | 0.572 | 0.552 | 0.562 | .565 |
| **Extra Trees** | 0.616 | 0.673 | 0.644 | 0.549 | 0.52 | 0.534 | 0.304 | 0.168 | 0.217 | 0.531 | 0.378 | 0.442 | 0.575 | 0.566 | 0.57 | .584 |
| **Neural Network** | 0.605 | 0.768 | 0.677 | 0.609 | 0.461 | 0.524 | 0 | 0 | 0 | 0.444 | 0.178 | 0.254 | 0.608 | 0.552 | 0.579 | .579 |
| **Engineered Single XGBoost Classifier** | 0.764 | 0.803 | 0.783 | 0.835 | 0.785 | 0.809 | 0.578 | 0.505 | 0.537 | 0.71 | 0.718 | 0.708 | 0.745 | 0.715 | 0.729 | .751 |
| **Engineered Hierarchical XGBoost Classifier** | 0.757 | 0.798 | 0.777 | 0.828 | 0.671 | 0.74 | 0.508 | 0.625 | 0.56 | 0.685 | 0.619 | 0.647 | 0.708 | 0.712 | 0.737 | .735 |

**Supplementary Table 1 : Tested models compared across various scoring metrics for each label**

**Figures of user interfaces and different clinical scenarios’ prediction**

**
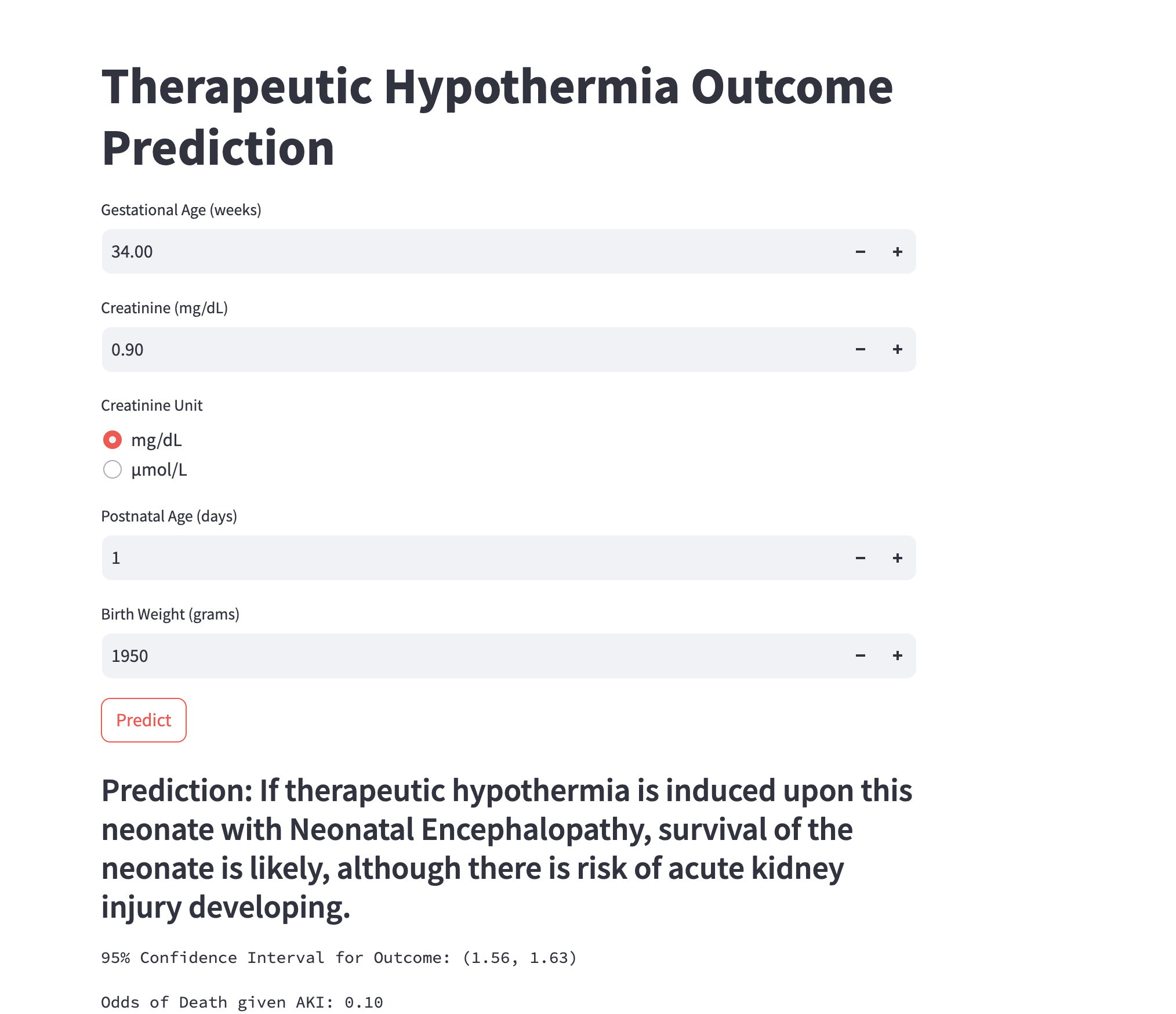
**

**Supplementary Figure 1: User interface of our developed model**


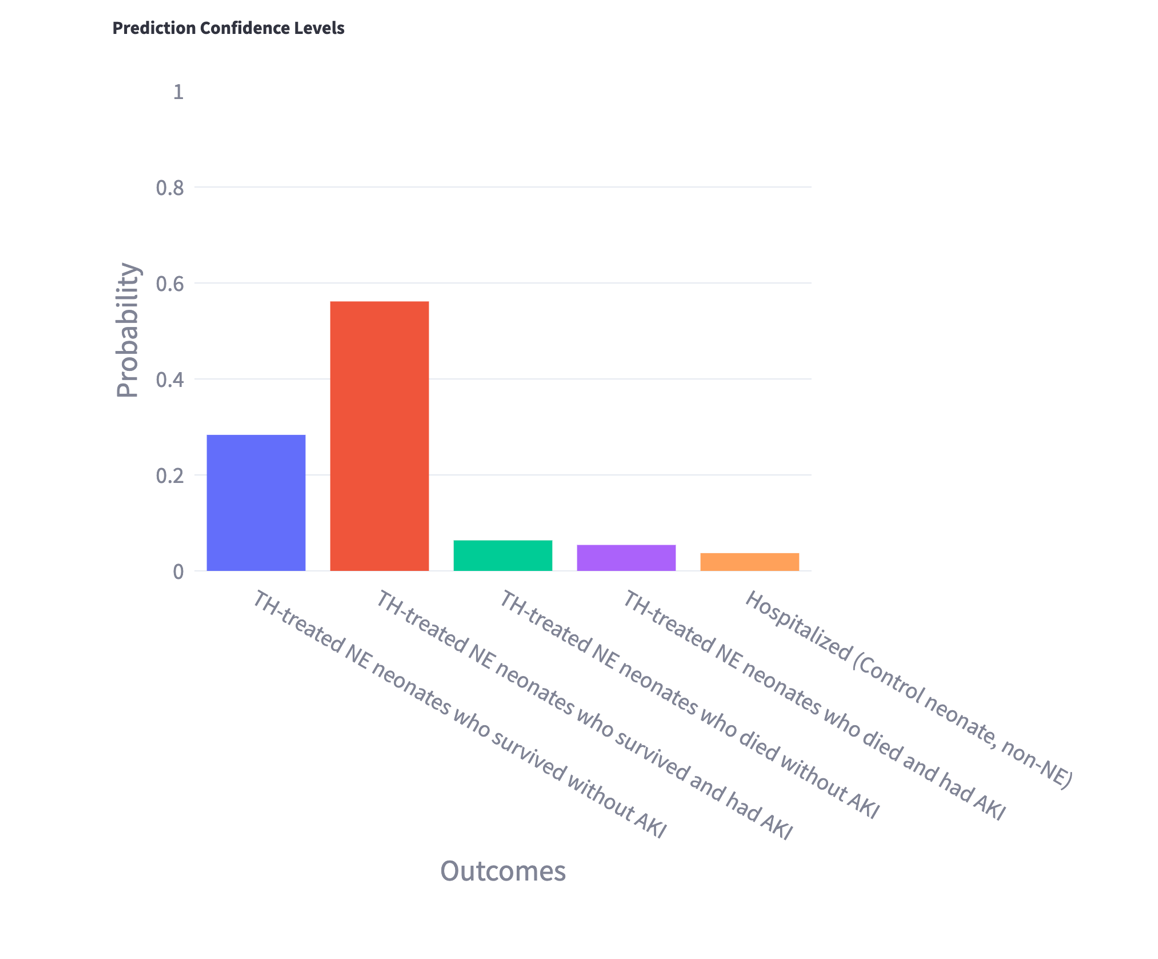
**
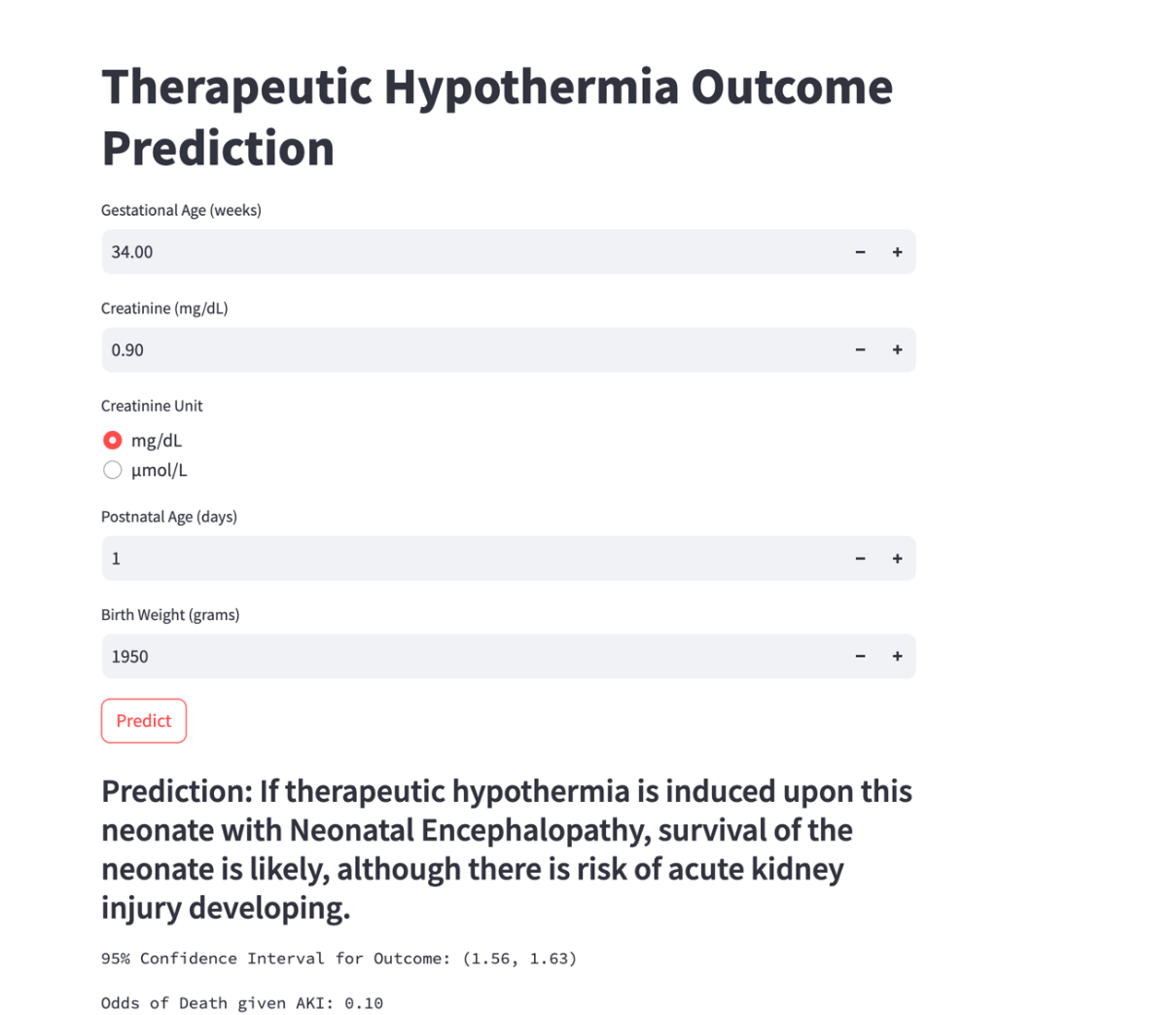
**

**Supplementary Figure 2: 34 weeks gestational age neonate on the first day of life**


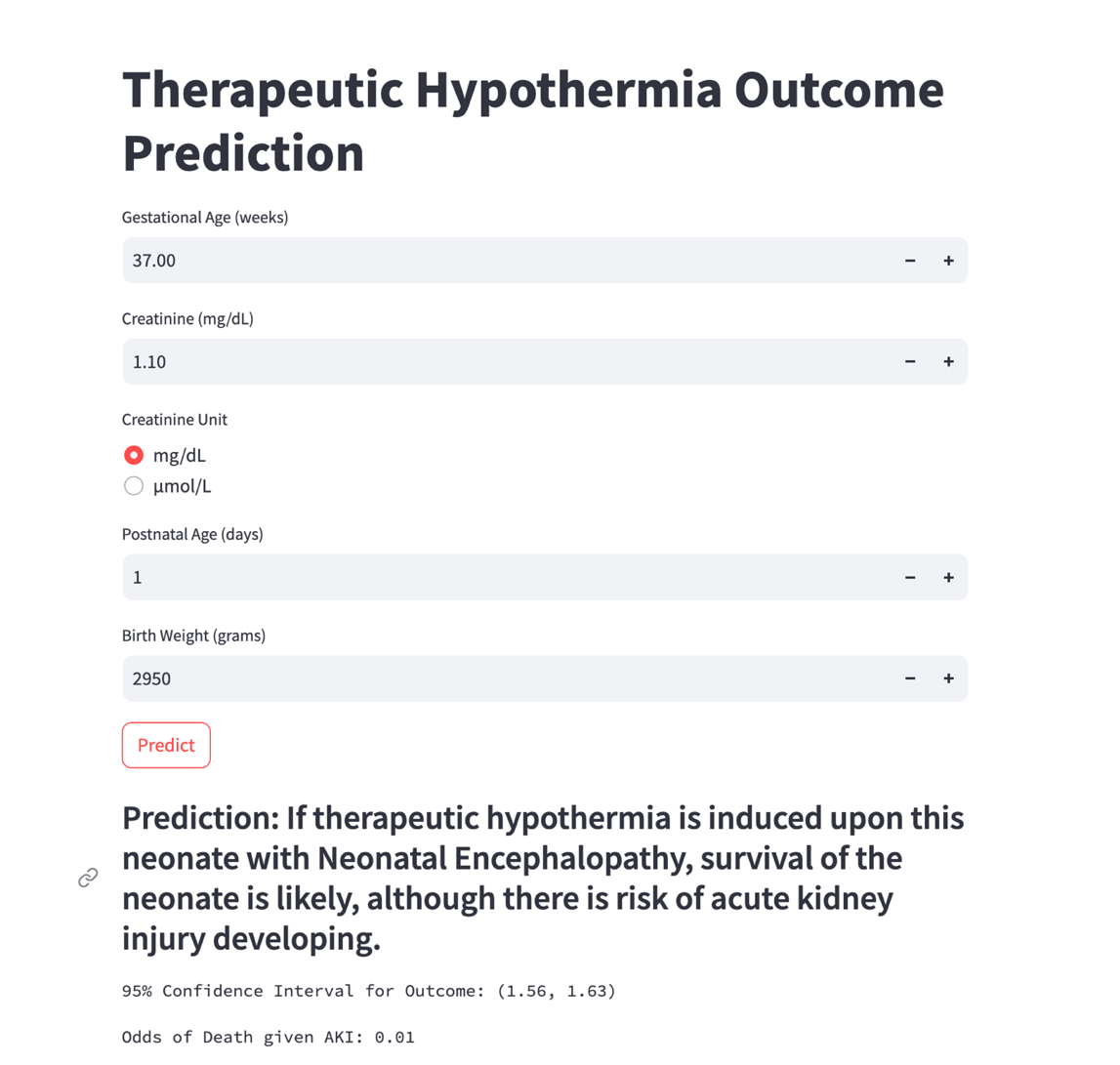

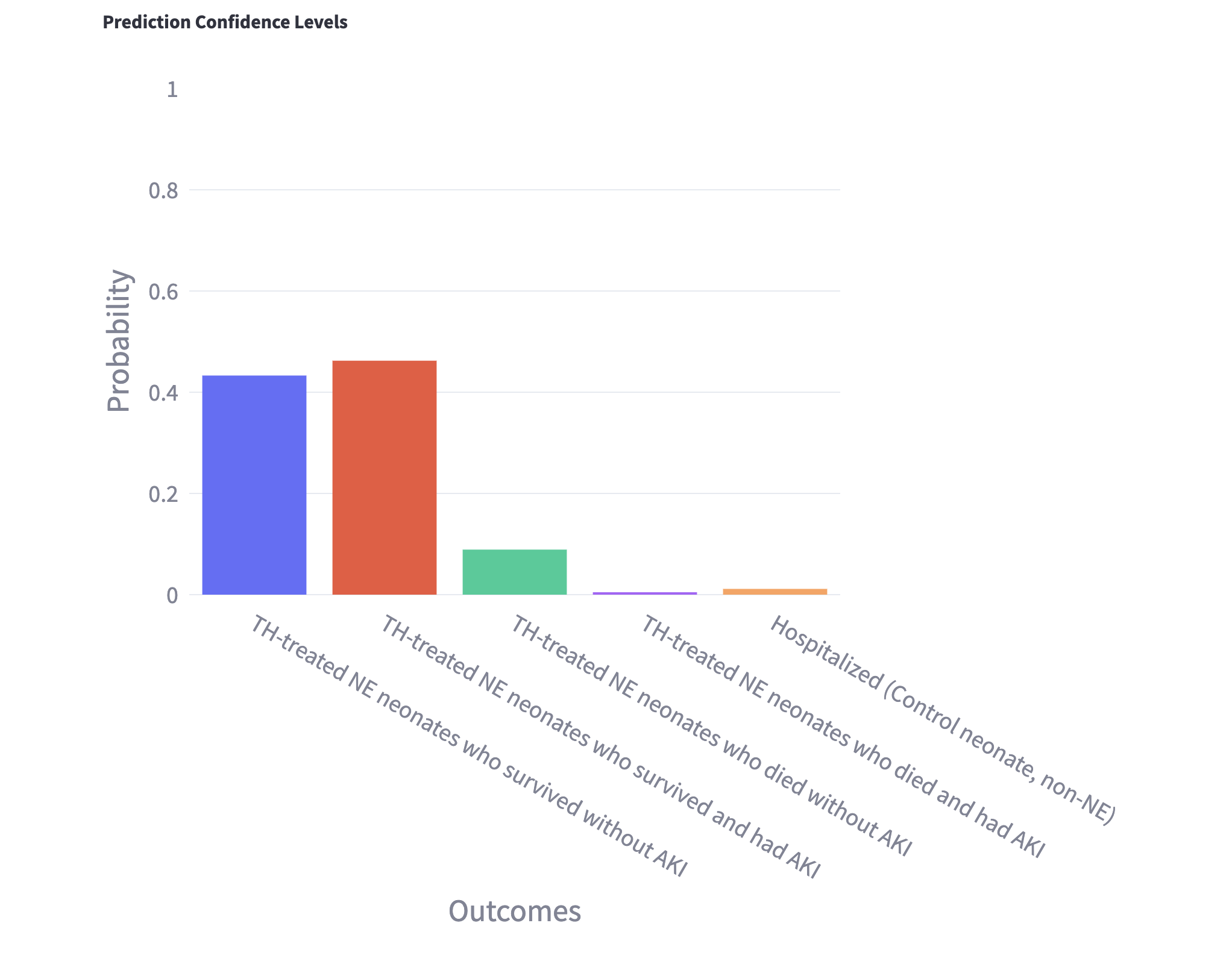


**Supplementary Figure 3: 37 weeks gestational age neonate on the first day of life**


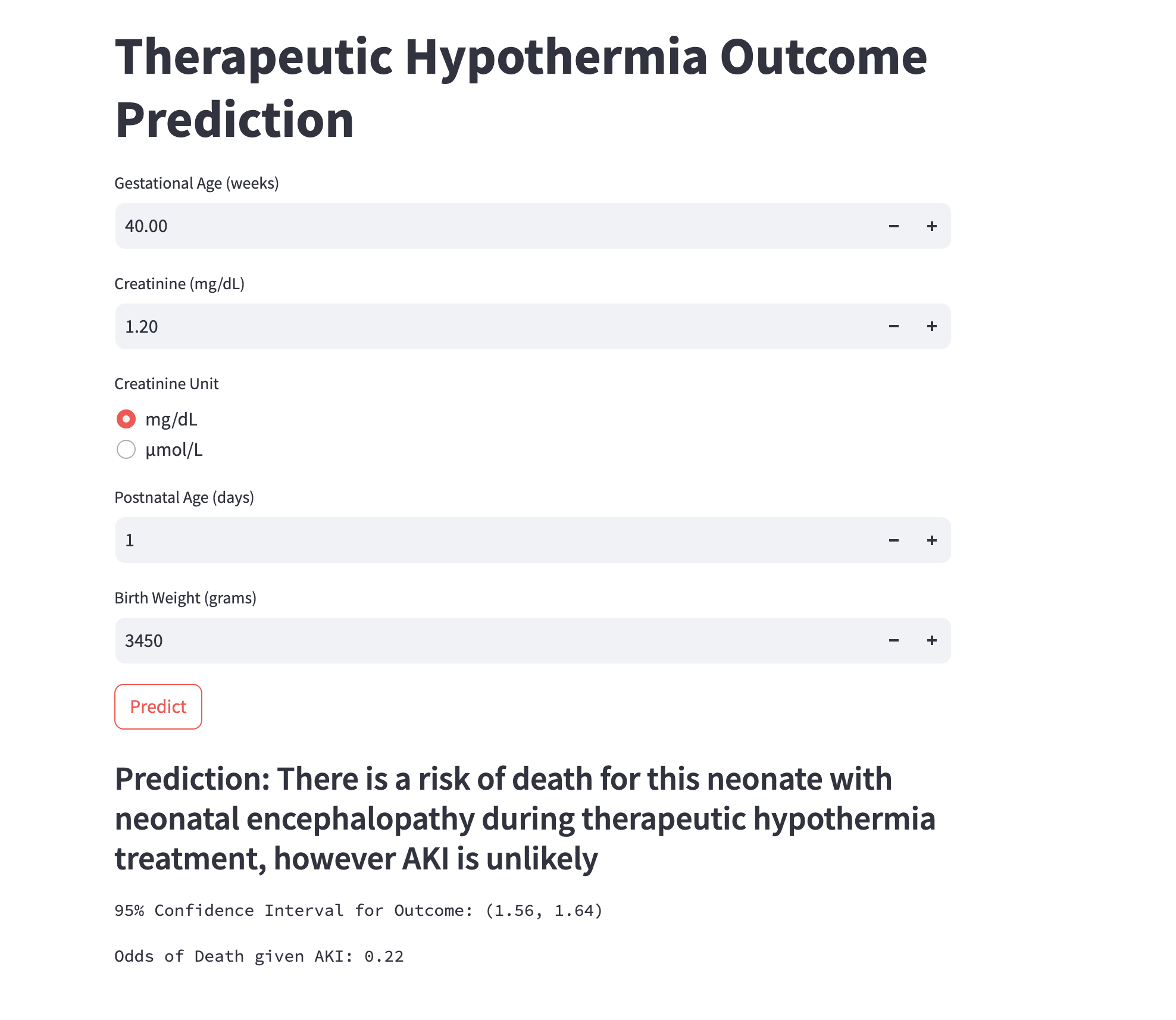


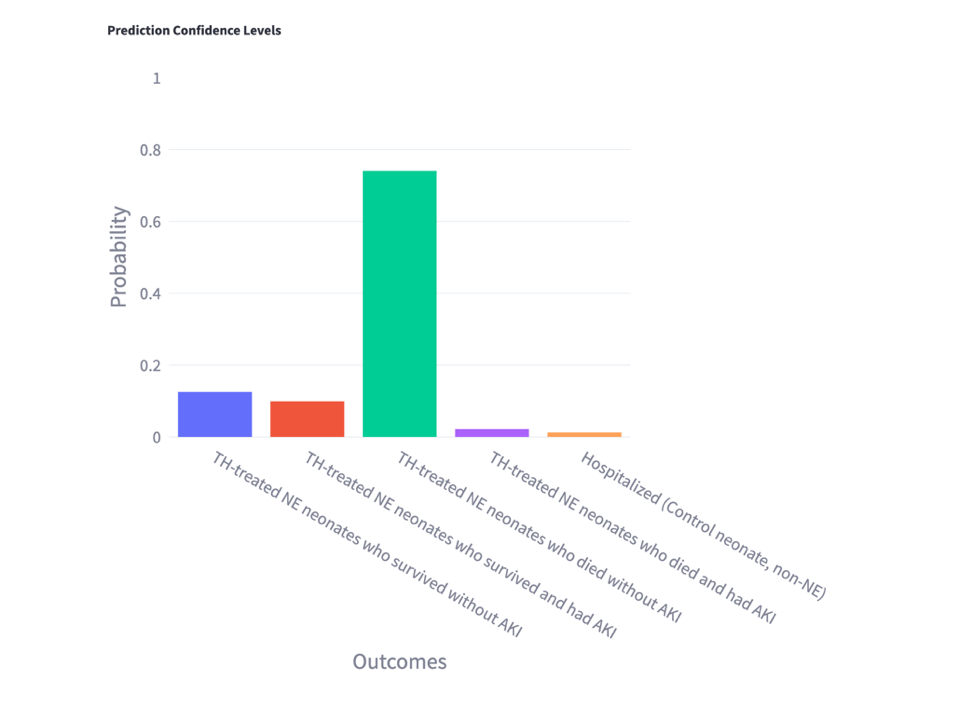


**Supplementary Figure 4: 40 weeks Gestational age neonate on the first day of life**


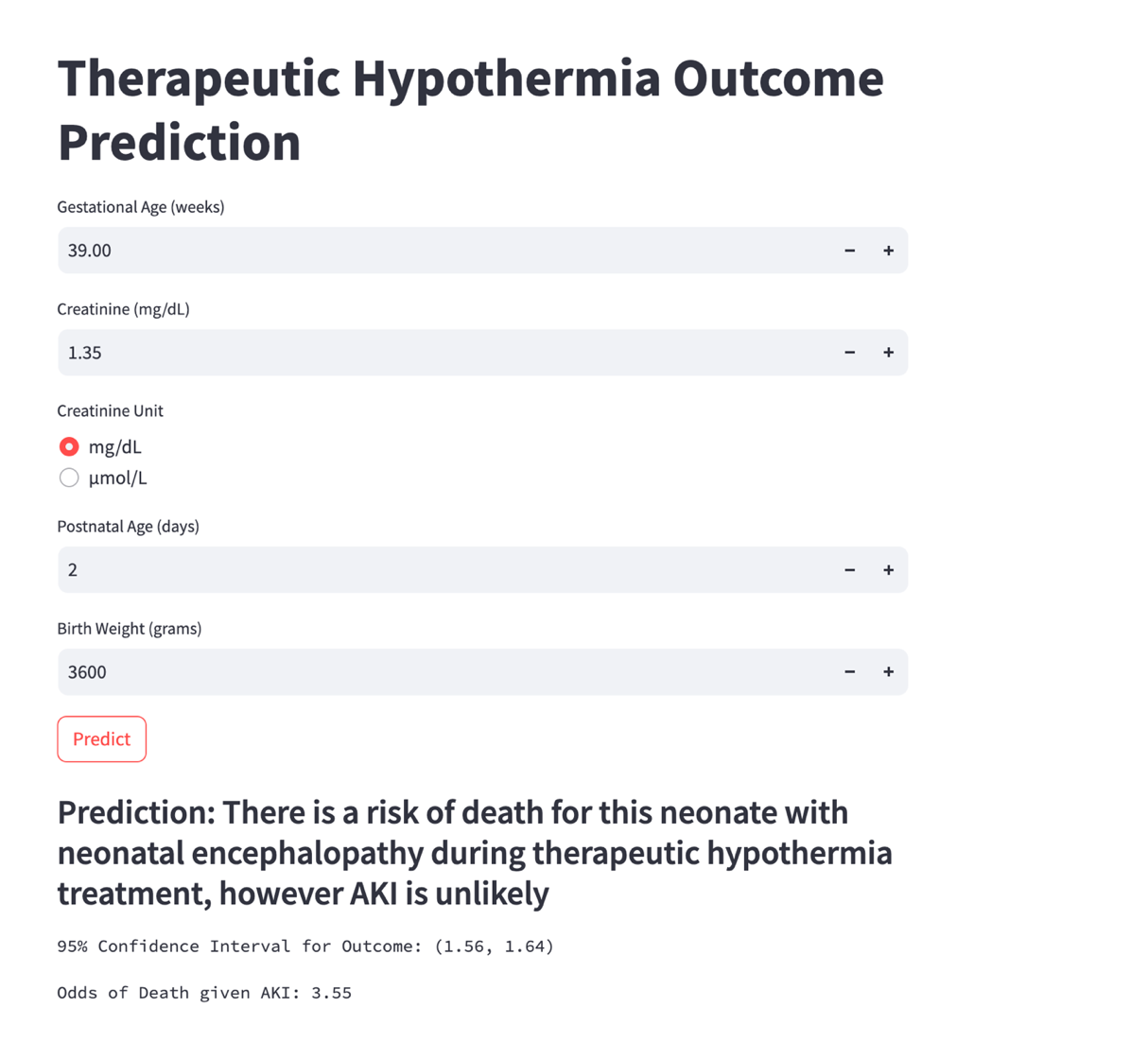


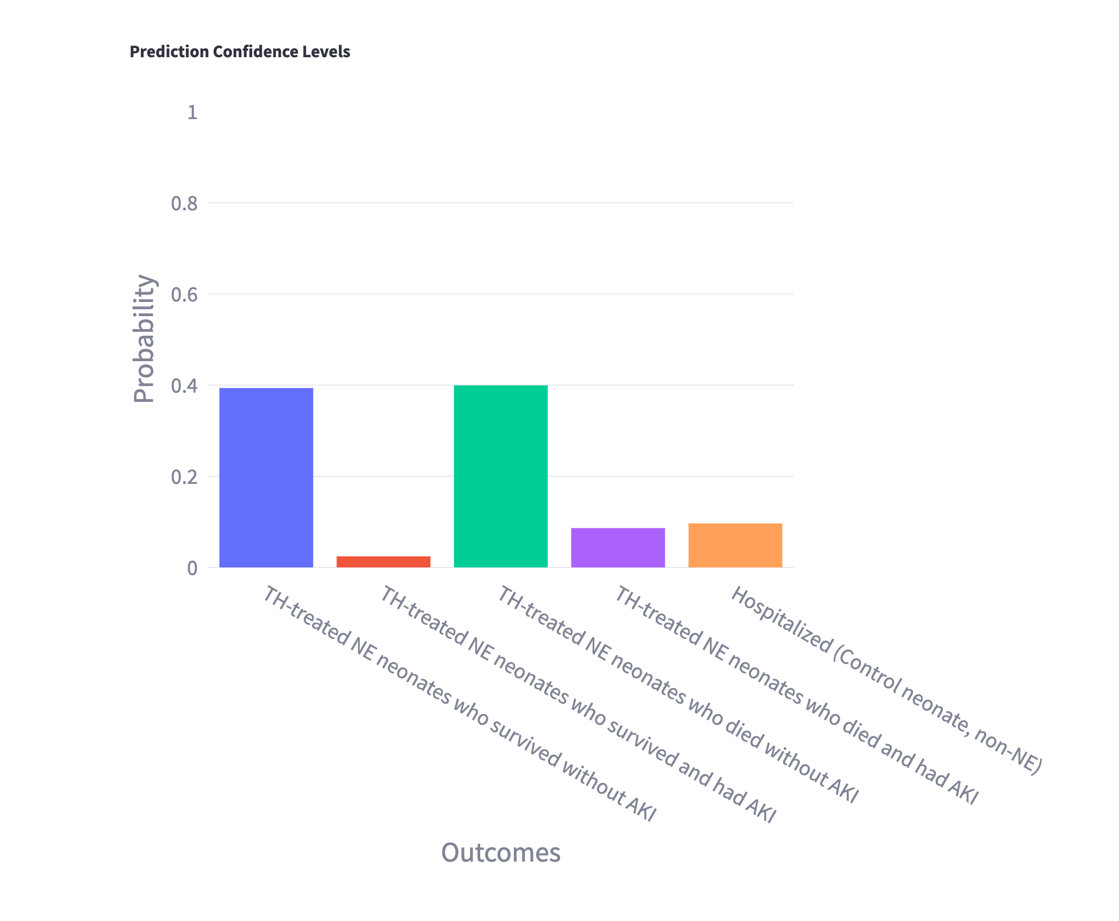


**Supplementary Figure 5: 39 weeks Gestational age neonate on the second day of life.**


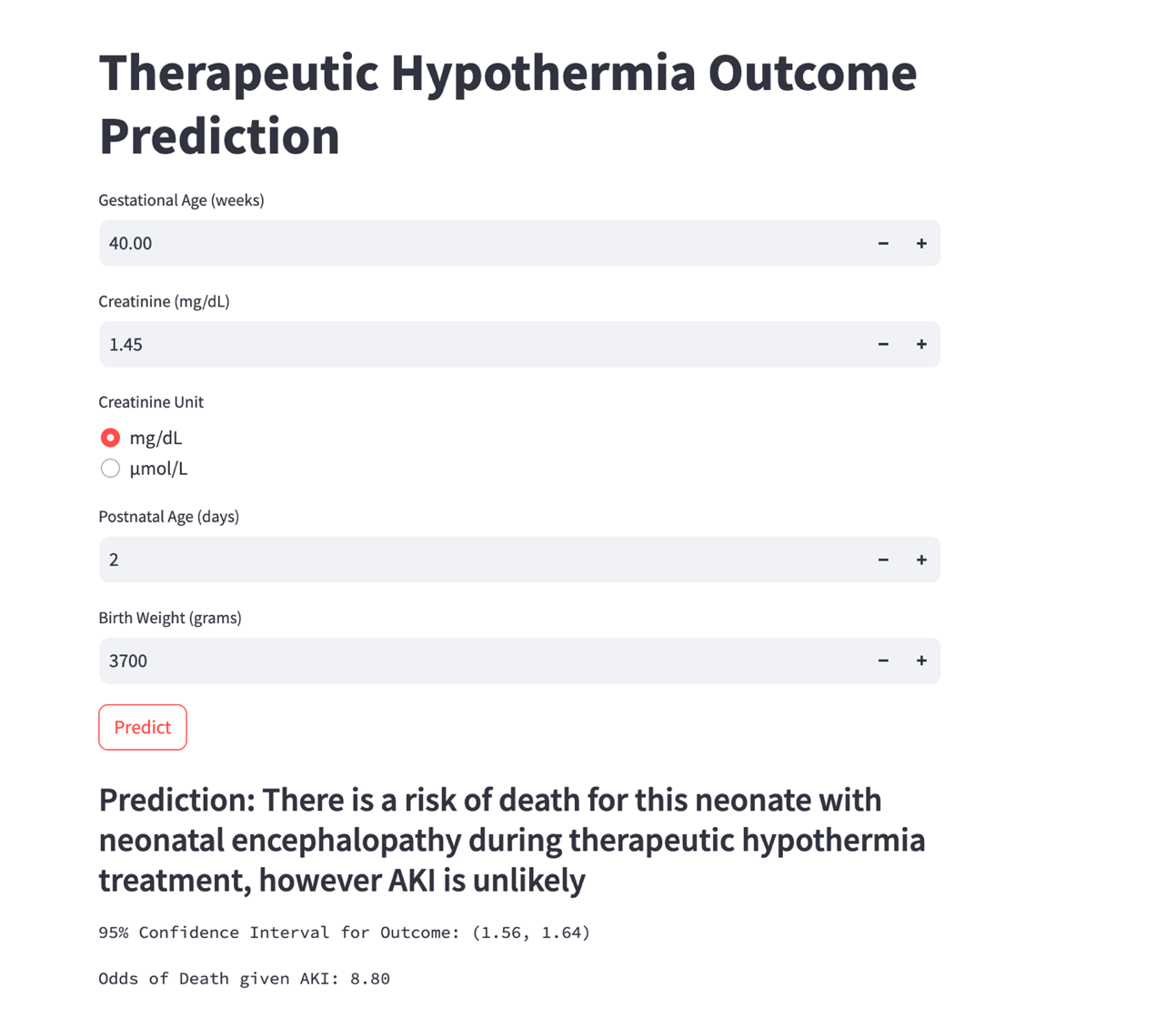


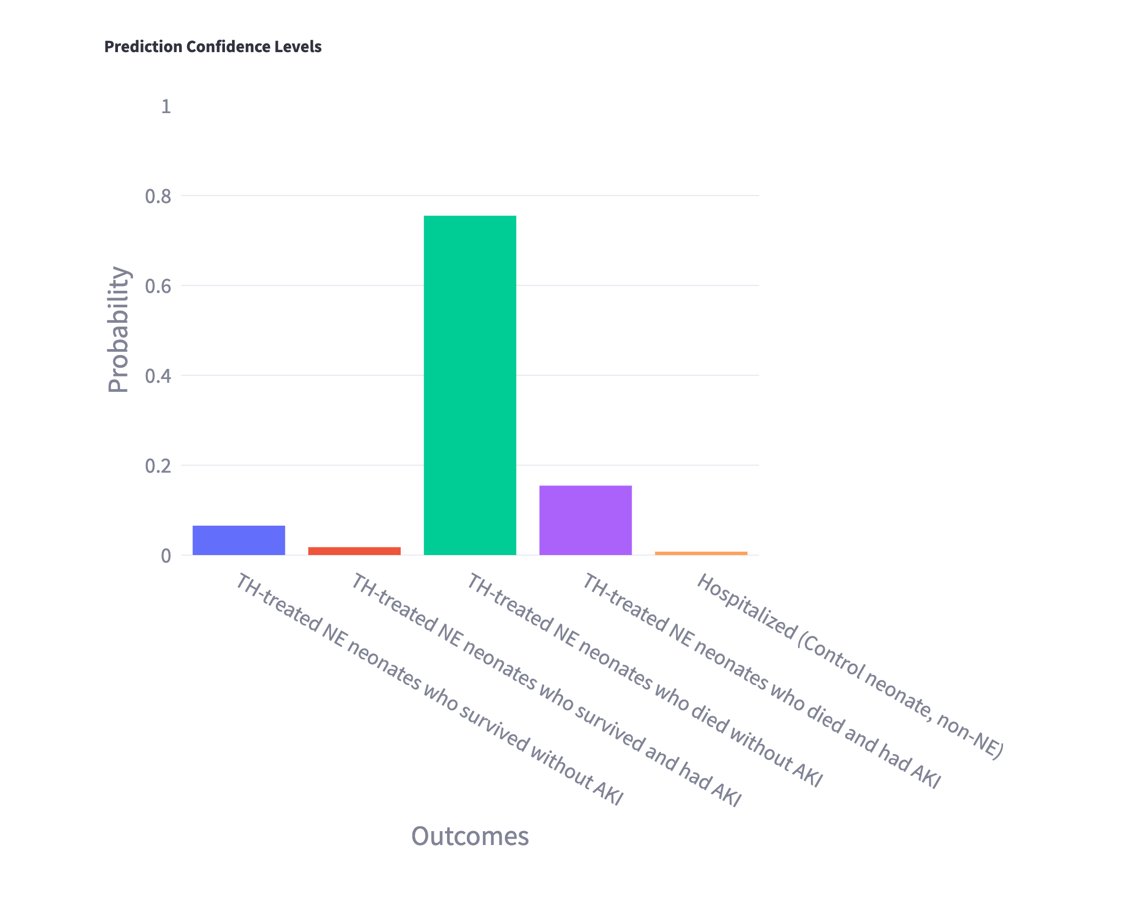


**Supplementary Figure 6: 40 weeks Gestational age neonate on the second day of life.**


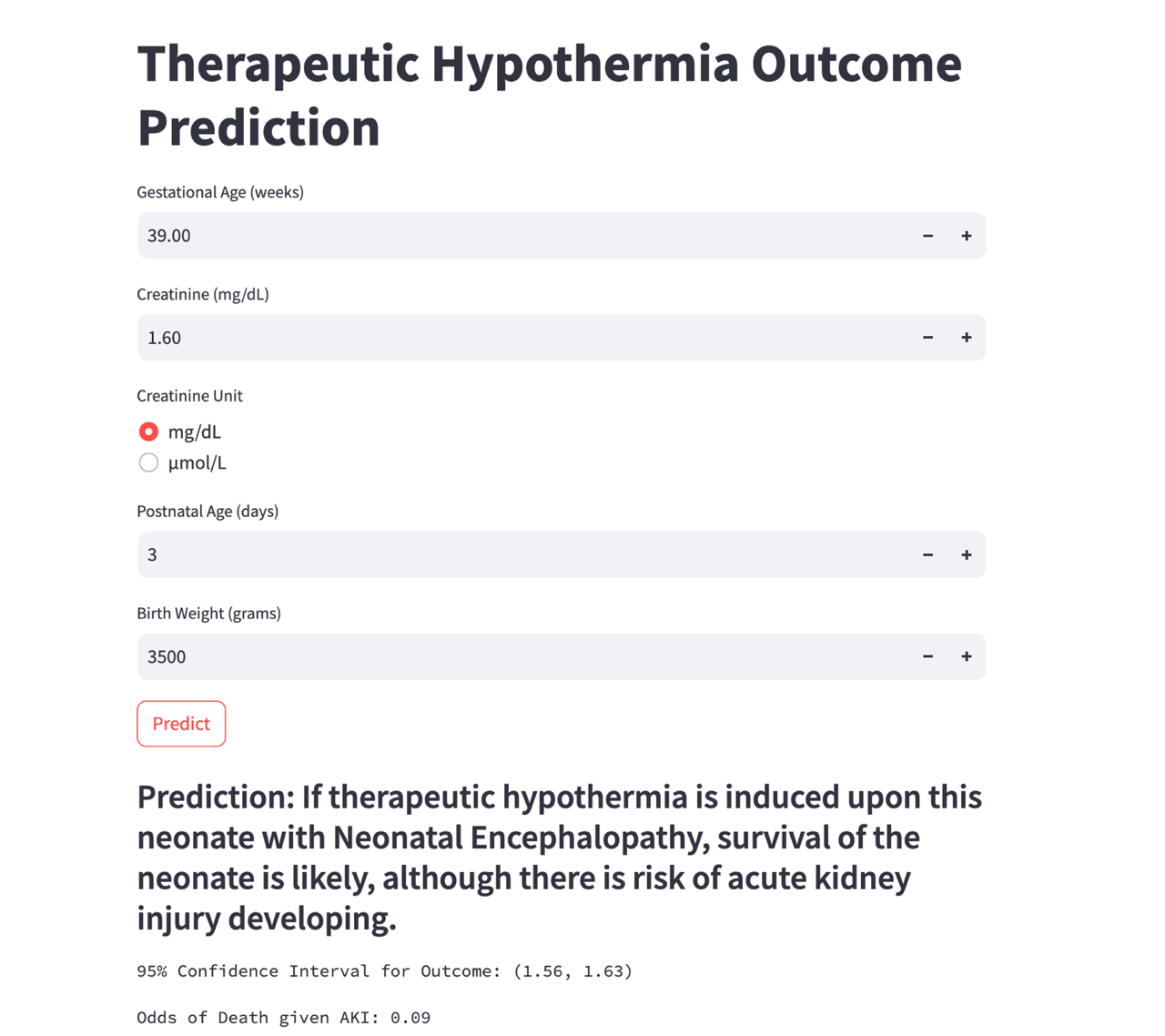


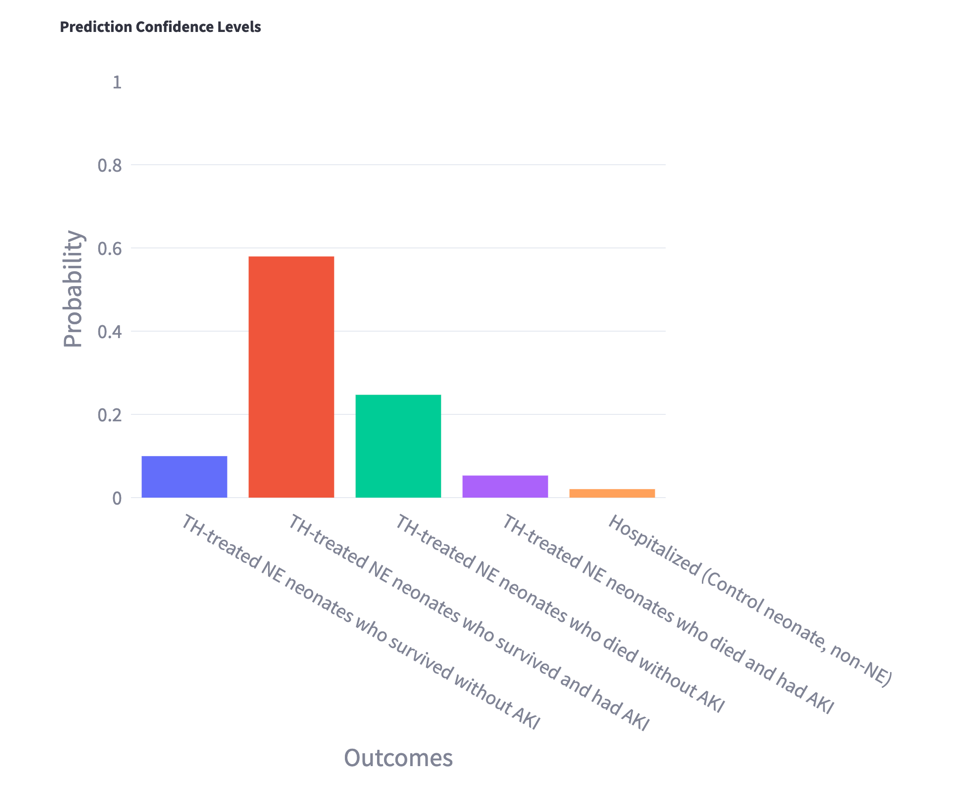


**Supplementary Figure 7: 39 weeks gestational age neonate on the third day of life.**


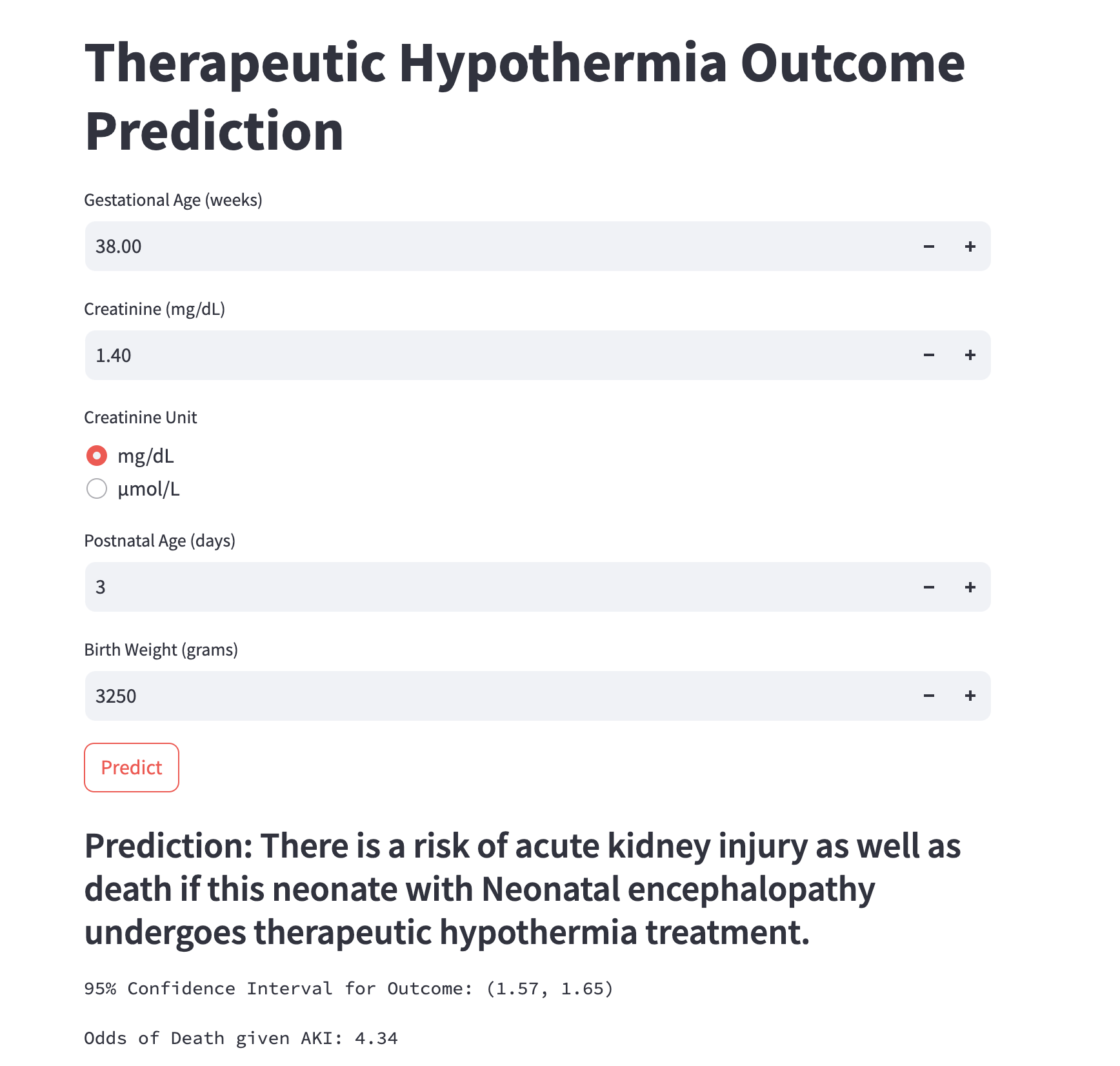


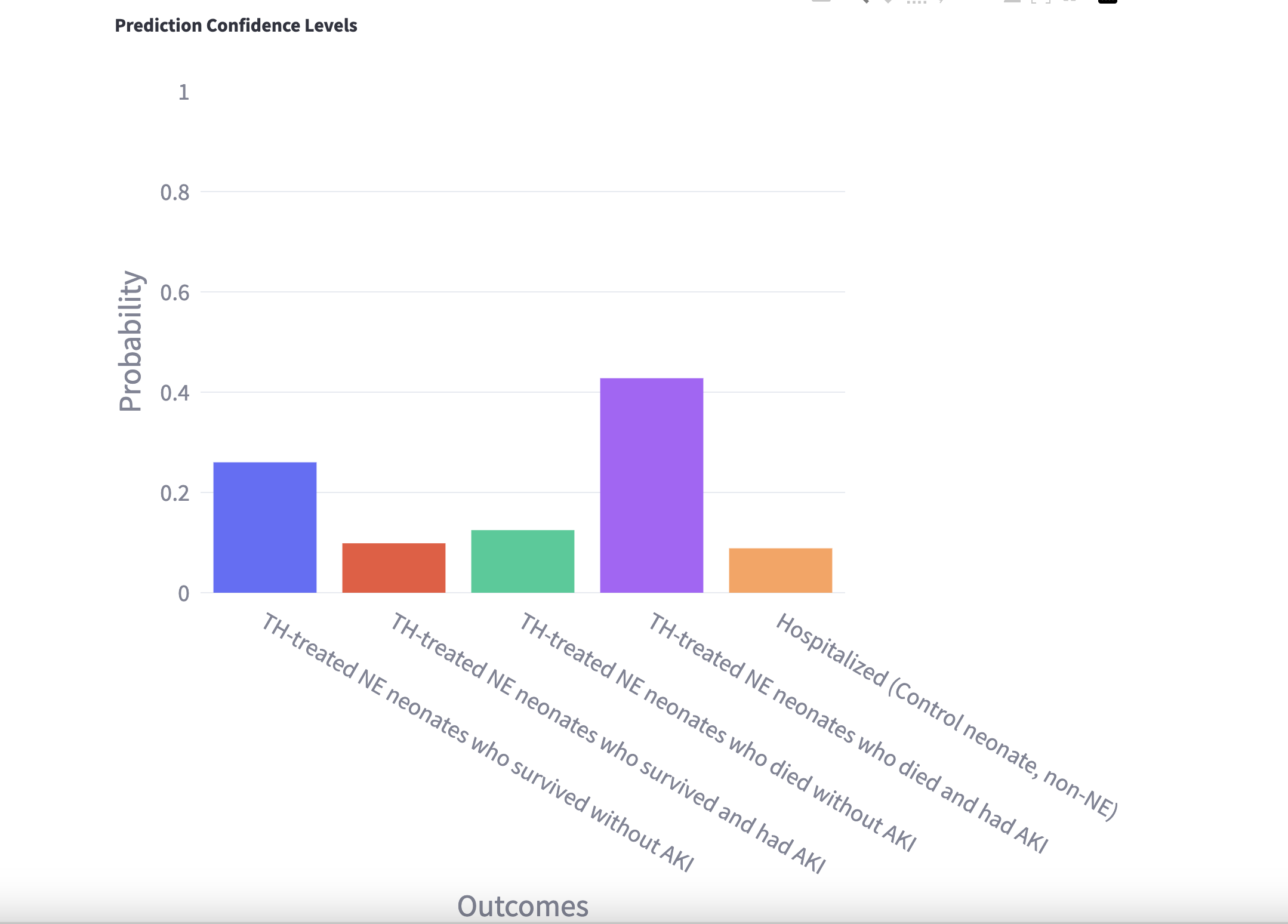


**Supplementary Figure 8: 38 weeks gestational age neonate on the third day of life**
